# A Novel Prediction Model for Brain Glioma Image Segmentation Based on the Theory of Bose-Einstein Condensate

**DOI:** 10.3389/fmed.2022.794125

**Published:** 2022-03-18

**Authors:** Tian Chi Zhang, Jing Zhang, Shou Cun Chen, Bacem Saada

**Affiliations:** ^1^School of Information Science and Engineering, Chongqing Jiaotong University, Chongqing, China; ^2^School of Information Science and Engineering, University of Jinan, Jinan, China; ^3^Shandong Provincial Key Laboratory of Network-Based Intelligent Computing, Jinan, China; ^4^Cancer Institute, Eighth Affiliated Hospital of Sun Yat-sen University, Shenzhen, China; ^5^Department of Animal Biosciences, University of Guelph, Guelph, ON, Canada

**Keywords:** image processing, brain glioma image segmentation, prediction model, quantum mechanics, Bose-Einstein Condensate

## Abstract

**Background:**

The input image of a blurry glioma image segmentation is, usually, very unclear. It is difficult to obtain the accurate contour line of image segmentation. The main challenge facing the researchers is to correctly determine the area where the points on the contour line belong to the glioma image. This article highlights the mechanism of formation of glioma and provides an image segmentation prediction model to assist in the accurate division of glioma contour points. The proposed prediction model of segmentation associated with the process of the formation of glioma is innovative and challenging. Bose-Einstein Condensate (BEC) is a microscopic quantum phenomenon in which atoms condense to the ground state of energy as the temperature approaches absolute zero. In this article, we propose a BEC kernel function and a novel prediction model based on the BEC kernel to detect the relationship between the process of the BEC and the formation of a brain glioma. Furthermore, the theoretical derivation and proof of the prediction model are given from micro to macro through quantum mechanics, wave, oscillation of glioma, and statistical distribution of laws. The prediction model is a distinct segmentation model that is guided by BEC theory for blurry glioma image segmentation.

**Results:**

Our approach is based on five tests. The first three tests aimed at confirming the measuring range of T and μ in the BEC kernel. The results are extended from −10 to 10, approximating the standard range to T ≤ 0, and μ from 0 to 6.7. Tests 4 and 5 are comparison tests. The comparison in Test 4 was based on various established cluster methods. The results show that our prediction model in image evaluation parameters of P, R, and F is the best amongst all the existent ten forms except for only one reference with the mean value of F that is between 0.88 and 0.93, while our approach returns between 0.85 and 0.99. Test 5 aimed to further compare our results, especially with CNN (Convolutional Neural Networks) methods, by challenging Brain Tumor Segmentation (BraTS) and clinic patient datasets. Our results were also better than all reference tests. In addition, the proposed prediction model with the BEC kernel is feasible and has a comparative validity in glioma image segmentation.

**Conclusions:**

Theoretical derivation and experimental verification show that the prediction model based on the BEC kernel can solve the problem of accurate segmentation of blurry glioma images. It demonstrates that the BEC kernel is a more feasible, valid, and accurate approach than a lot of the recent year segmentation methods. It is also an advanced and innovative model of prediction deducing from micro BEC theory to macro glioma image segmentation.

## Introduction

### Background

In brain image segmentation, the input brain image is provided, and the precision of the segmented object contour output may be judged by manual observation. However, the segmentation procedure between the input and output stage is not a fixed process. It may be based on predicting or applying a theory borrowed from other research fields. For example, several experimental studies show that the predictions used in the transfer process from input to output are correct when they are associated with the brain image segmentation. In that case, we propose a new prediction model based on the theory of the Bose-Einstein Condensate (BEC) to improve the process of segmentation.

The BEC is a quantum ground state discovered by Bose and Einstein describing the statistical distribution of bosonic atoms when cooling to a shallow temperature (1). This external temperature is called the quantum critical point and is associated with a divergence of particle density (2). The quantum necessary is different from the crucial classical point because as the temperature decreases, density increases, and more particles are forced into a single state (3). In addition, BEC has five characteristics: (1) it does not behave independently, (2) has dynamics properties of atomic condensate, (3) exhibits a change of the symmetry with the gas density, (4) collapses into a single state, and (5) may be described by a single and uniform wave function (4). In BEC applications, most researchers have focused on testing the dynamic processes of stability and phase transition, the change of symmetry, wave function, etc. (5–12). For example, Rajagopal and Muniandy (13) focused on the dynamic processes of atomic condensate to couple with the distinct dual reservoirs. Guo and Li (14) describes symmetry and topology in a fractional non-linear Schrodinger system by applying a wave function. In a change of symmetry application, Haag et al. (15) used symmetry to analyze the processes of pumping and absorption in optical waveguide systems. Tsatsos and Lode (16) applied the change of the symmetry of gas density to describe the appearance of resonances, that is, peaks in the total energy appeared when the stirring frequency was increased. In summary, the analysis of symmetry and wave function has led to a wide range of applications in many technical and scientific research fields. Here we take another BEC-important feature of the dynamic processes of stability and phase transition to offer essential characteristics in terms of image processing that differ from the classical techniques used before. It raises a crucial question: could the dynamical processes of stability and phase transition of the BEC help us propose a novel prediction model or method, especially in challenging application of brain image segmentation?

### The Concept of BEC and Glioma

**Figure 1:**
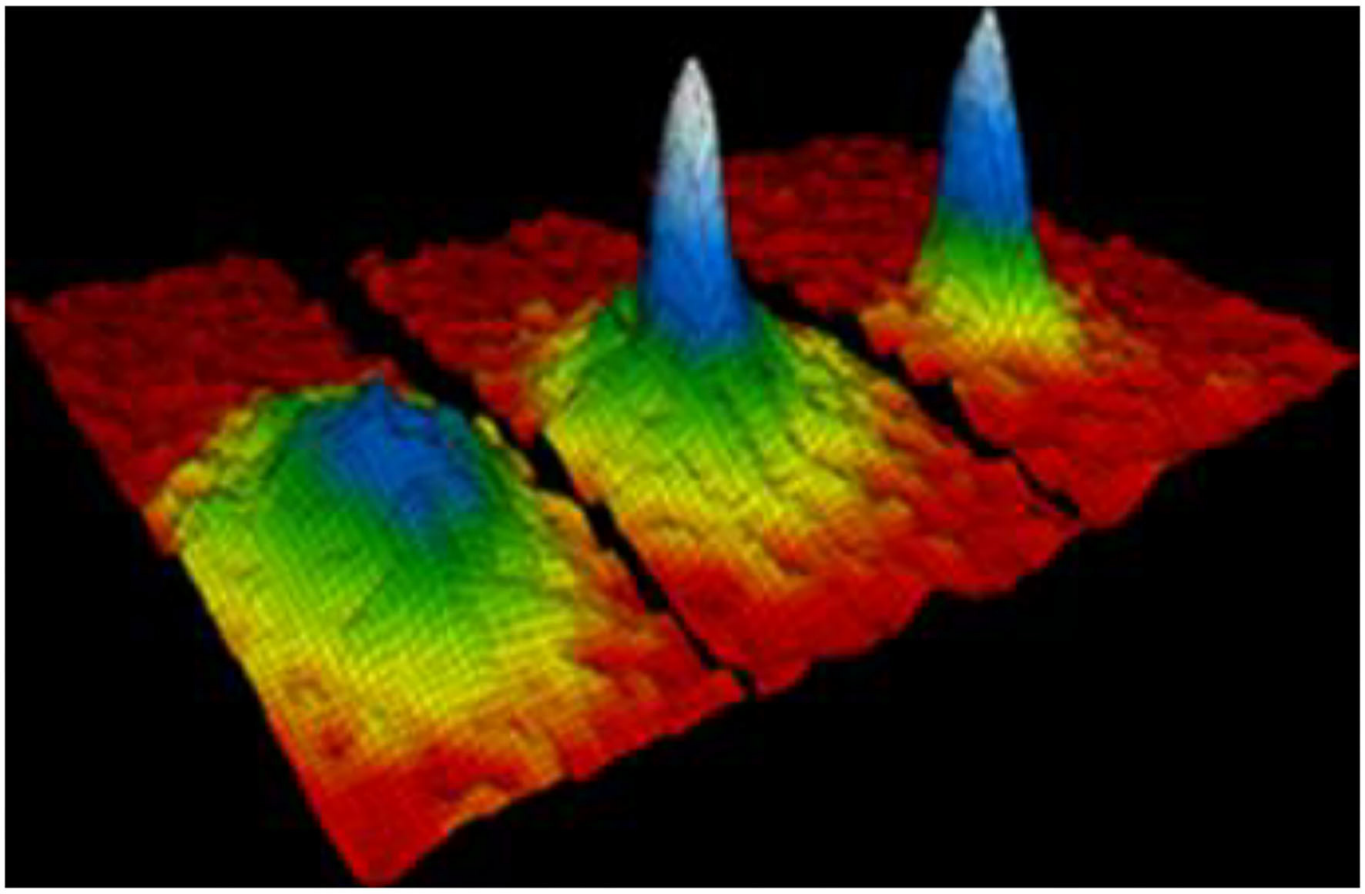
Bose-Einstein Condensate (BEC).

Bose-Einstein Condensate (BEC) is a macroscopic quantum phenomenon that is also called a dynamic single matter state. When a Bose gas is cooled to temperatures close to absolute zero, such as near 0(K) or −273.15(°C), more and more bosons atoms are moved into one lowest quantum state (17). The BEC motion state was predicted by Satyendra Nath Bose and Albert Einstein in 1924. As illustrated by Figure 1 (18), the left displays the state before the appearance of a BEC, while the center shows the state after the condensate formation, and the right indicates the state of a nearly pure condensate. This reveals that BEC is composed of three phases. The colors in Figure 1 indicate the number of particles at different speeds of orbit. In the beginning of BEC condensation, some particles are in the green region, represented by the low-speed orbit. During the condensation process, the number of particles decreases in the blue region, representing them at the lower speed orbit. When the condensation is formed, all particles condense into a single particle in the light blue region of the lowest speed orbit. A peak on the right image indicates that the BEC effect exists, while no peak on the left indicates that the BEC effect does not exist. The velocity distribution can be given by the width of the trough in the curvature formula. The smaller the width of the trough is, the closer it approaches the BEC condensed state. Therefore, based on both the color of peak and shape of the trough, we can determine the stage of BEC condensation.

Glioblastoma is the most common malignant brain tumor, with about 40–60%. The average survival time of glioma patients is 6–9 months (19). The image shape characteristic of a glioma is an unclear contour with, at best, a visible cystic or ring enhancement (20). According to recent animal studies, along with limited epidemiological evidence, radiofrequency radiation leads to an increased risk of brain glioma, especially in the case of mobile phone addiction or long-term cell phone use (21). It is because radiation with low energy can influence the wave frequency of electrons outside the nucleus and the binding force between particles, such as atoms and ions (22).

#### The Similarity Between BEC and Glioma

Glioma is one type of tumor that is caused by irritant factors, such as radiation and nuclear, chemical, and microbial pollution. In the following section, we will analyze the similarity between the BEC and glioma according to two respective causes: a group comprised of nuclear, chemical, microbial pollution, and another comprised of electromagnetic radiation.

In mainstream quantum theory, the BEC has considered the experimental proof of wave function collapse. It demonstrates that the eigenstates of superposition collapse into a single matter state. The quantum mainstream theory of superposition collapse is relevant to microscopic particles. The human body is composed of tiny particles that have a system of wave functions (23). According to the Grand Unification Theory (GUT), the decay of light rays is similar to wave function collapse (24). The destruction of glioma, caused by radiation, also focuses on the wave function collapse, as described by the BEC (25, 26). For example, when atoms are approaching absolute zero, they move much slower than at average temperatures. David Hudson has speculated that the characteristics of a brain tumor at natural temperatures have very close features to that at absolute zero (27–29). Consequently, a glioma may cause the formation of the BEC at room or higher temperatures.

In addition, an artificial synthetic cell might be considered as a quantum-based electron molecular, with self-assembly and metabolism change according to quantum electron excitation and tunneling equations (30–32). In that case, the charge transfer in the simulated cell may be viewed as a quantum particle-wave trace (33). Living organisms under nuclear, chemical, and microbial pollution might be simulated by quantum mechanical theory (34). Tumor cells are abnormal in metabolism, structure, and function. Their proliferation is greater than that of normal cells. The hyperplasia of some tumor cells is thought to be caused by irritant factors (35), such as nuclear, chemical, and microbial pollution. Tumor cells might have similar properties to the artificial cells, and so might be simulated by quantum mechanical theory. Therefore, we postulate that the image segmentation of glioma can benefit from applying the principles and theory of quantum mechanics, such as in the case of the BEC.

**Figure 2:**
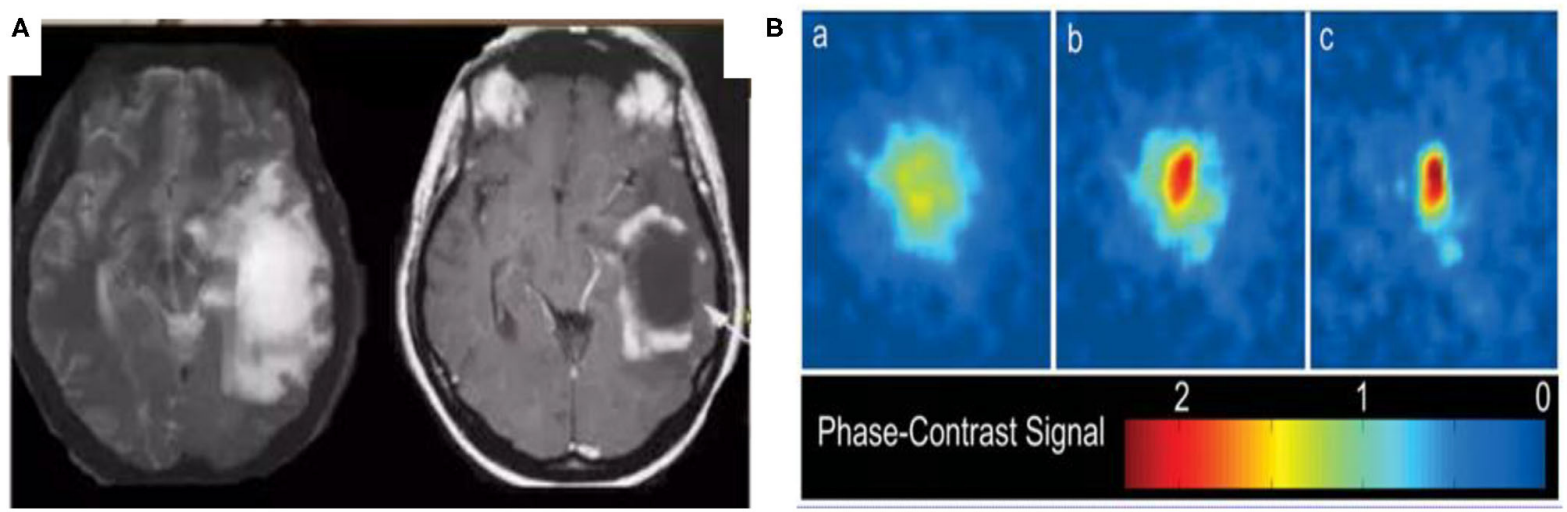
Similarities between glioma and BEC. **(A)** glioma of T2W1, **(B)** 2D image of BEC. a: before the appearance of a BEC. b: just after the appearance of the condensate. c: nearly pure condensate.

In the following section, we investigate the similarities between glioma and the BEC through the analysis of their shape. Figure 2A is a typical image of glioma^1^ and Figure 2B is a 2D image of a BEC^2^. We can immediately see that the shape of the glioma is similar in appearance to that of the BEC. For example, all atoms tend to stick together when a BEC of a million atoms is achieved under a shallow temperature. When a one million atom BECs at the lowest temperature, all atoms tend to stick together. According to the formula of quantum mechanics, the shape of BEC condensation process can be expressed by an equation. Similarly, due to irritant factors, if brain cells cannot balance growth and division, a glioma may develop into a shape similar to that of the BEC. Therefore, we predict and assume that the glioma shape might have the same quantum mechanics formulation as that of the BEC.

Figure 2 highlights similar properties in the shape of Bose-Einstein Condensate (BEC) and glioma.

In the next section, we introduce our research on representing the image segmentation prediction model for glioma using BEC theory.

## Methods

Based on the similarity between BEC condensation and glioma in appearance contour, this section explains the principle and formula of BEC condensation to propose a contour segmentation model that describes glioma. It proves the similarity and representability between BEC condensation and glioma formation in accordance with relevant theories.

In this section, first we study the formulation of the BEC and propose a prediction model of brain image segmentation using a BEC kernel. Second, we discuss the relationship between the BEC kernel and quantum mechanics and analyze the relationship between the BEC kernel and the glioma features. Third, we validate the BEC kernel approach using a Laplace distribution and by Poisson distribution. Finally, we affirm the advantages of the prediction model with the BEC kernel.

**Figure 3:**
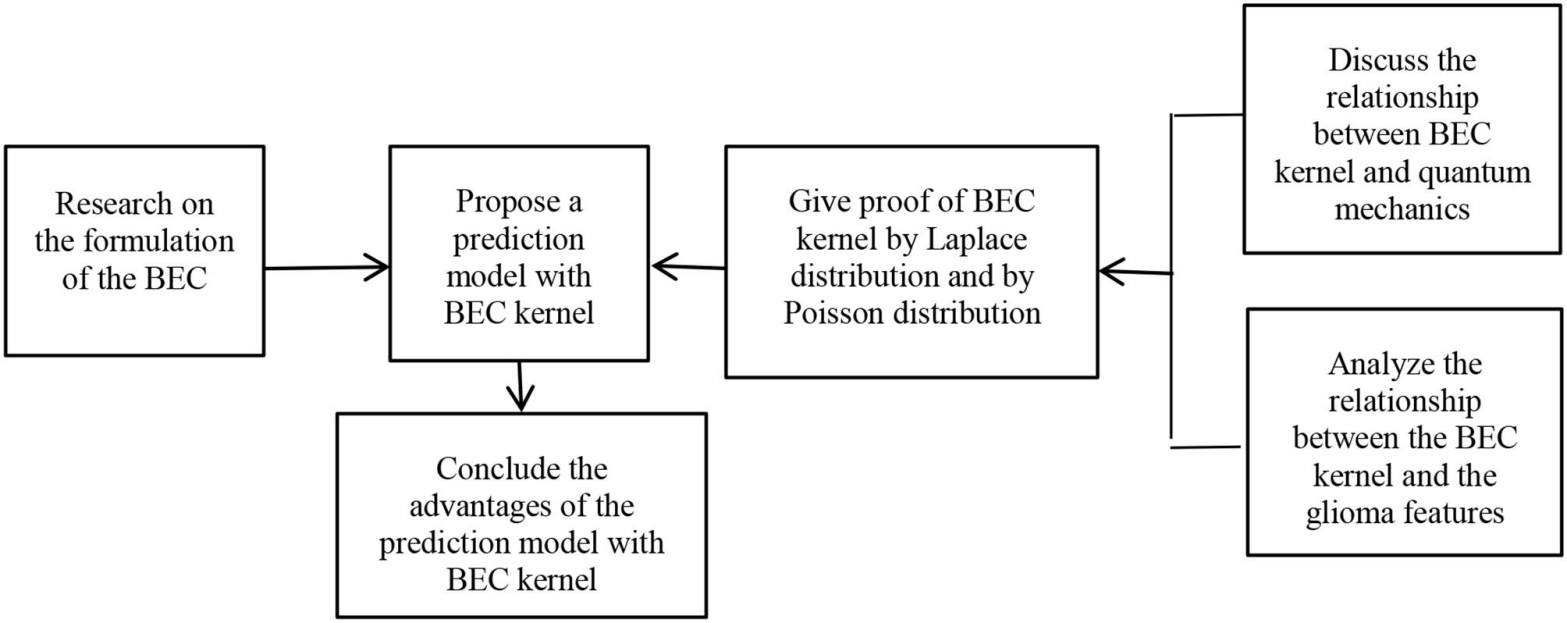
The framework of the proposed method.

The framework of the proposed method is shown in Figure 3.

### The Formulation of BEC

The BEC forming condition means that the Boudreau wavelength of the particle should exceed the distance between the particles. Here, Bose-Einstein statistics are used to describe the equilibrium state that any ideal gas should obey. The critical temperature and critical particle density might be determined by the equation below^3^. We assume that there is a perfect gas composed of N bosons in the container of volume V when N bosons are in balance; they satisfy the equation of Bose-Einstein statistics:


(1) $$\begin{array}{l} \text{n}\left(\varepsilon_{i}\right)=\frac{1}{\exp^{\left[\left(\varepsilon_{i}-\mu \right)/k_{B}T\right]}-1} \end{array}$$

where n(ε_*i*_) represents the number of particles with ε_*i*_ energy in a balanced state. μ gives chemical potential, μ ≤ 0 and K_B_ represents Boltzmann's constant, K_B_ = 1.3806488 × 10^−23^ (J/K). T is the temperature of particles and T_c_ is the quantum critical temperature, T_c_ = 0(K) or −273.150(C). If there is T ≤ T_c_, the BEC state occurs.

The BEC formula above is described by a square one negative exponential function, and the square two exponential functions express the Radial basis function (Gaussian) kernel for image segmentation. It raises the question of whether there are some relations between the two processes. The next part discusses the two functions and proposes the prediction model for brain image segmentation.

### The Prediction Model With the BEC Kernel

Generally, the segmenting hyper-plane in image segmentation is often represented by a separating kernel, such as the Radial basis function (Gaussian) kernel or S kernel function (36). The Gaussian kernel is a variant of the mathematical approach, expressed by the square two exponential function, as shown below:


(2) $$\begin{array}{l} G\left(x_{1},x_{2}\right)=\exp\left(\frac{{\left||x_{1}-x_{2}-\mu |\right|}^{2}}{2\sigma^{2}}\right) \end{array}$$

Typically, the variance σ = 0.125 and μ is the expected value. Compared with Formula (1), we know that the distinction between BEC and Gaussian functions is the number of the power in the exponent. Particles of ε_*i*_ might instead be represented by a node pixel *x*_1_ − *x*_2_ in the glioma image, so Equation (2) can be rewritten as follows and named our BEC kernel:


(3) $$\begin{array}{l} G\left(x_{1},x_{2}\right)=\frac{1}{\exp\left(\frac{\left||x_{1}-x_{2}-\mu |\right|}{K_{B}T}\right)-1} \end{array}$$

Two super-parameters might be changed in the BEC kernel, T and μ. The parameter T refers to the BEC temperature. Its value in image segmentation can be determined after experimentations. T value may be negative, while its suggested range is from 0 to 10 based on the degree of BEC occurring. The lower T value is, the more the phenomenon of the BEC appears. The other parameter, μ, might be decided by experiment, and its range is suggested to be from 0 to 7 based on BEC physical features.

Similarly, for our BEC kernel for brain image segmentation, we use this prediction model because the kernel arises from the process of BEC formulation, which is a natural principle inside a quantum mechanical theory that reveals the forming state in microscopic particles, including the living cells. The BEC kernel is raw and foresighted, and we believe it indicates and predicts an utterly novel model. As a macroscopic world model, we omit the magnitude of values in Formula (3) of 1.38 × 10^−23^ by replacing it with 1.38.

The prediction model of brain image segmentation is:


(4) $$\begin{array}{l} G\left(x_{1},x_{2}\right)=\frac{1}{\exp\left(\frac{\left||x_{1}-x_{2}-\mu |\right|}{1.38\times \left(T\right)}\right)-1} \end{array}$$

where *x*_1_ − *x*_2_ indicates the position of a node. Initially, T is the temperature of the particle and μ represents a chemical potential. Here, in image segmentation, T and μ are parameters determined according to the type of image. The prediction model with the BEC kernel is the critical contribution of this article. The differences between the kernels we proposed by using the BEC and Gaussian are: (1) the quantum-mechanics theory informs the BEC kernel function of the BEC. It follows from a similar forming process, both for the glioma cell and BEC. The proposed BEC kernel function is novel, predictive, and fits more with the laws of nature than the Gaussian kernel. (2) The BEC is an exponential function (1nth power, Linear) instead of an exponential function (2nth Power) of the Gaussian kernel. It means that our method has a lower time complexity and higher efficiency. It is required for any image processing algorithm to be as efficient as possible. Therefore, the execution time should be reasonable. It should be polynomial and not factorial.

The detailed running steps and the algorithm of a prediction model with the BEC kernel in image segmentation are as follows:

(1) Input an original medical image
(2) Convert the points pixel (m, n) of the original image into a one-dimensional matrix (x_1_, x_2_, ……)
(3) Use BEC kernel function: $\text{G}\left({\text{x}}_{1},{\text{x}}_{2}\right)=\frac{1}{\exp\left(\frac{\left||{\text{x}}_{1}-{\text{x}}_{2}-\;\mu \;|\right|}{1.38\times \left(\text{T}\right)}\right)-1}$ to train the dimensional matrix
(4) Classify (x_1_, x_2_,……) matrix into (0, 0, ……) and (1, 1, ……) parts
(5) Output the part of the (0, 0,……) as the background and the part of (1, 1, ……) as the target region.

### The Relationship Between BEC Kernel and Quantum Mechanics

For over 40 years, quantum mechanics, especially string theory, has been considered an ideal candidate to represent the laws of nature, including the composition of materials and life itself (37). For example, Wei Tong, a Fields Medal winner, said, “All the great ideas of physics are the by-product of superstring theory” (38). This is based on the proposition that all around us is composed of tiny vibrating strings and not particles (39). So, vibrating strings and superstrings are the core theory of quantum mechanics.

**Figure 4:**
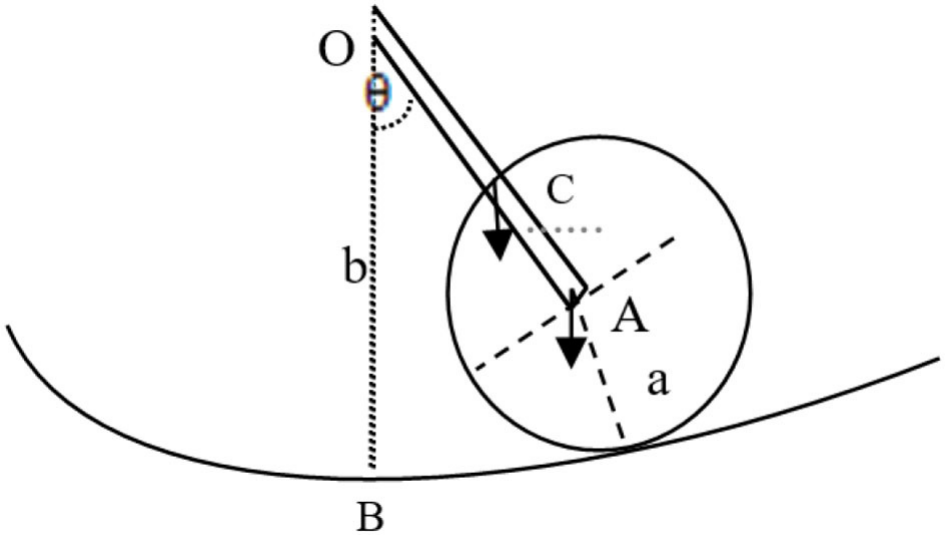
The components of a pendulum.

In essence, the vibrating string appears like the movement of a pendulum (40). The vibrating pendulum shown by Figure 4 includes a mass m1 connected with m2 with a straight rod and radius A. The other end of that rod is fixed in space and associated with the center O of a cylinder with radius B, where the rod length is l = b–a. Thus, the disk vibrates around point O. The equipment takes the point B as the standard potential energy V:


(5) $$\begin{array}{l} \text{V}=\text{g}\left(\text{b}-\text{a}\right)\left({\text{m}}_{1}+\frac{{\text{m}}_{2}}{2}\right)\left(1-\cos\;\theta \;\right) \end{array}$$

The formula above shows that string vibration can be presented by an equation that includes the function of cos θ, namely, a string vibrating move in the form of a cosine wave.

With the development of the string vibration theory, some essential features of moduli stabilization are represented by the KKLT (Kachru-Kallosh-Linde-Trivedi) model (41). First, the KKLT moduli are installed in a de Sitter vacuum, for which the super-potential energy of KKLT is:


(6) $$\begin{array}{l} W=W_{0}+Ae^{aT},|W_{0}\ll 1| \end{array}$$

where W_0_ is flux and rendered constant, the *a* indicates the mass, and T is the Käahler modulus. Thus, string theory, as described by the KKLT moduli, is coupled. Being composed of a solid interacting hidden sector of proportion A, and a tree-level Kahler potential, K = −2 ln (Tb + Tb^*^) 3/2, the scalar potential energy of KKLT moduli is formed as:


(7) $$\begin{array}{l} \text{V}\left(\text{t}\right)=-{\text{a}}^{2}{\text{A}}^{2}{\text{e}}^{-2\text{at}}/6\text{t} \end{array}$$

The equation above shows that a formula that includes the negative exponent can present quantum mechanics movement.

In summary, quantum mechanics theory can be used to represent the laws of nature, including the law of life, and it can be described in the form of a cosine wave or function of a negative exponent. The BEC kernel includes a negative exponent, as is also the case for the KKLT model. The BEC kernel with its negative exponent could also represent nature's laws, especially for the natural law of life.

### The Relationship Between the BEC Kernel and the Glioma Features

Several exciting questions now follow: Do material waves and glioma have some common relationships? What are the internal motions and deformations of materials? The different properties of the material are generalized by other waves and might be represented by the different wave equations (42). To solve these problems, physicists create various wave equations according to the various internal structures. High-frequency waves can “order” nuclear spins to a specific oscillation. Usually, particles of all physical nature behave like waves, and a wave function describes their state. The wave function of a dimensional harmonic oscillator is


(8) $$\begin{array}{l} \psi \left(\xi ,\text{t}\right)=\sum_{n}\alpha_{n}e^{-\xi^{2}/2}{\text{H}}_{n}\left(\xi \right)e^{i\left(n+1/2\right)\omega t} \end{array}$$

A wave function with the oscillator above indicates the role of a plural exponent. The latter may take the form of a Hamiltonian with the self-adjoint operator in quantum mechanics. All the eigenfunctions within Hamiltonian have the characteristic of complete orthogonal basis (43). Defined in range (0, 2π), the eigenfunction f (x) is:


(9) $$\begin{array}{l} \text{f}\left(x\right)=\sum_{n=0}a\cos\text{nx}+b\sin\text{nx} \end{array}$$

The factor of e^∧^i[(n+1)/2]ωt in Equation (8) might be written as:


(10) $$\begin{array}{l} e^{i\left(n+1/2\right)\omega t}=\cos\left(n+\frac{1}{2}\right)\omega x+isin\left(\left(n+\frac{1}{2}\right)\omega x\right) \end{array}$$

There is a connection between Equations (9) and (10) as of e^∧^i[(n+1)/2]ωt and f(x) = acos(nx) + bsin(nx), because that cos(nx) + bsin(nx) is the real part as a plural of e^∧^i((n+1)/2)ωt. Both can represent the oscillation movement of particles. Our previous research patent on the protein folding process proved that f(x) = acos(nx) + bsin(nx) could represent the oscillation of protein folding in a cell (44). Matthew Fisher et al. noted a quantum coherence on macroscopic time scales while small molecules and ions rapidly entangle in a surrounding wet environment (45). Nuclear spins in molecules are also weakly coupled to their environmental degrees of freedom. However, in the case of brain cells, there is a prolonged phase coherence (46). The complex quantum wave function includes a real part, the magnitude of the amplitude, and the phase of the imaginary part. It is analogous to a negative exponential function.

We have a wave function graph of e^∧^i((n+1)/2)ωt about y-axis symmetry with e^∧^(–i((n+1)/2)ωt) and the BEC kernel as:


(11) $$\begin{array}{l} G\left(x_{1},x_{2}\right)=\frac{1}{\exp\left(\frac{\left||x_{1}-x_{2}-\mu |\right|}{1.38\times \left(T\right)}\right)-1} \end{array}$$

We can conclude that the BEC kernel is similar to the wave function. However, the BEC can represent a change of energy, emphasizing the process of changing. The BEC kernel also has a relationship with the cell oscillation formula, where the BEC kernel can describe a more complete and comprehensive cell oscillation.

The relationship between the uncertainty of quantum mechanics and the macroscopic world: one problem is the probabilistic logical causal chain, and the other one is the logical causal chain. To bridge these two kinds of causal chains, it is necessary to normalize the probabilistic logical causal chain. The normalized quanta can be regarded as the complete quanta or set of quantum groups which are deterministic, causal, knowable, and described by macroscopic laws. The unnormalized quantum can only be regarded as a fragment of the complete quantum, or a transient fragment representation of the complete quantum, which is uncertain, random, and unknowable, following the description of the causal chain of probabilistic logic.

The following are the theoretical proof sections. The Laplace distribution equation and the Poisson distribution equation are the spatial distributions of natural laws proved by probability. They both involve the negative exponential factor of 1, similar to our BEC kernel.

### The Proof of BEC Kernel by Laplace Distribution

Initially, when Laplace invented his Laplace distribution, he wanted to see the law of nature as created by God (47–49). Here we ask, in spirit, or organism of glioma: Is there an underlying law with a distribution that the oscillation of a cell obeys? In probability theory and statistics, the Laplace distribution, named after Pierre-Simon Laplace, is composed of exponential distributions. A Laplace (μ, b) is a random variable, and the Laplace distribution function is:


(12) $$\begin{array}{l} f(x|\mu ,b)=\frac{1}{2\text{b}}\text{exp}(-\frac{\mu -x}{b})\\ \;=\frac{1}{2\text{b}}\{\begin{array}{ll} \text{exp}(-\frac{\mu -x}{b}) & \text{ if }x<\mu \\ \text{exp}(-\frac{x-\mu }{b}) & \text{ if }x\geq \mu \end{array} \end{array}$$

where μ is a location parameter and b is a scale parameter sometimes referred to as the diversity, b >0. The BEC formula is: $\exp{\left(\frac{\left||x_{1}-x_{2}-\mu |\right|}{1.38\times \left(T\right)}\right)}^{-1}$. It has Y-axis symmetry with $\exp{\left(\frac{\left||x_{1}-x_{2}-\mu |\right|}{1.38\times \left(T\right)}\right)}^{}$, so the BEC formula is similar to Laplace distribution of the equation, $\frac{1}{2b}\exp\left(-\frac{\left|\mu -x\right|}{b}\right)$.

In summary, the Laplace distribution represents a natural law, and it helps explain a principle of nature, including living cells, such as glioma. Our BEC kernel describing internal structure of glioma is similar to Laplace distribution.

### The Proof of BEC Kernel by Poisson Distribution

The Poisson distribution is also a theory in probability and statistics. It is named after Siméon Denis Poisson, a French mathematician. It describes an average rate law in a given space or time, with the condition that there is a given number of events within a fixed interval. It can also be extended in several disciplines for distance, area, or volume, and can even be used as a random process model in the fields of astronomy, biology, ecology, geology, physics, economics, image processing, and telecommunications (50). In our space representation of image segmentation, the Poisson distribution has its advantages and can express a law describing a spatial distribution. The probability of nodes in an image for a Poisson distribution is as follows.

Assume that nodes occur interval is 0, 1, 2, … The node rate as the average number is designated Lambda λ and the probability of observing node k in an interval is:


(13) $$\begin{array}{l} \text{P}\left(\text{k events in interval}\right)=\frac{\lambda^{\text{k}}{\text{e}}^{-\lambda }}{\text{k}!} \end{array}$$

Where e is Euler's number, 2.71828…, it is the base of the natural logarithms. The Poisson distribution is better in representing a space probability distribution than a Normal distribution (51). The equation for Poisson distribution is similar to the equation of Laplace distribution, and they both include the factor-negative exponent which is identical to the BEC kernel. Together, they help prove that the BEC kernel is feasible and reasonable in theory, given the negative exponent. The BEC kernel, thus, has its theoretical basis both in quantum mechanics and natural law as mathematics and statistical distribution.

In summary and based on the theory of quantum mechanics, statistical distribution, and related research, this section proves that the BEC kernel is suitable for glioma image segmentation.

### The Advantages of Our Prediction Model With BEC Kernel

This article has three advanced and innovative research highlights. First, an advanced concept of predictive segmentation is proposed. This model is not only a brain glioma image segmentation model, but is also a prediction model, which predicts the contour of a blurry medical image that cannot easily be segmented. Second, the theoretical proofs deduce the relationship between the micro theory of quantum mechanics and macro glioma contours by using the micro formation mechanism of a brain tumor to help the macro glioma segmentation. Based on the quantum mechanics, the novel BEC prediction kernel is the third frontier or research highlight of this article. The detailed prediction models with BEC kernel are: (1) the brain belongs to nature and is composed of molecules and ions. The theory of quantum coherence and nuclear spins can express a law describing the formation of a brain tumor, including that of glioma. Glioma forming might be defined according to the theory of quantum coherence and nuclear spins; that is, we can use quantum coherence and nuclear spins, such as BEC and its equation, to describe the forming process of glioma. Therefore, the application of BEC theory to glioma segmentation has a theoretical basis. (2) Small molecules and particles have quantum coherence in the micro-world. However, in the macro world, the nuclear spin of small molecules and particles is weakly coupled with the degree of freedom of the environment. The nuclear spin is very weak and can be easily changed by the environment. It is in a way similar to the etiology and evolution mechanism of glioma.

We, therefore, conclude that the BEC kernel may represent not only a law of nature but may also be used to analyze the images of glioma usefully.

## Experiments

We performed five tests to investigate whether our proposed BEC kernel with Support Vector Machine (SVM) can be used to implement glioma image segmentation tasks. Test 1 aimed to cluster nodes within a segmented set, and Test 2 considered the same nodes within two groups. Test 1 and Test 2 confirmed that the BEC kernel is effective in doing single or multi-classifications and in realizing a segmented shape of nodes that are similar to glioma formation. Test 3 investigates if the BEC kernel is feasible for glioma image segmentation using 18 glioma images divided into different types. Test 4 examined the performance of the BEC kernel by comparing it against other existing cluster methods in the literature. The images used in the tests are selected both from the database^4^ and from reference papers. Finally, test 5 aimed to do a further test compared with the existing Convolutional Neural Networks (CNN) methods using the challenging Brain Tumor Segmentation (BraTS) datasets and clinical images for segmentation.

### Test 1: Nodes in One Set

Test 1 explored the ability of the BEC kernel to cluster image nodes. In this test, one set of random nodes is classified into two distinct classes in two dimensions. One type of node formed a circular central area and an annular region of a different radius. By clustering using the BEC kernel and the Gaussian kernel, we investigated whether the shape of the nodes clustered by the BEC kernel appeared similar to the natural form of glioma and whether it had a better ability than the Gaussian kernel to cluster nodes.

**Figure 5:**
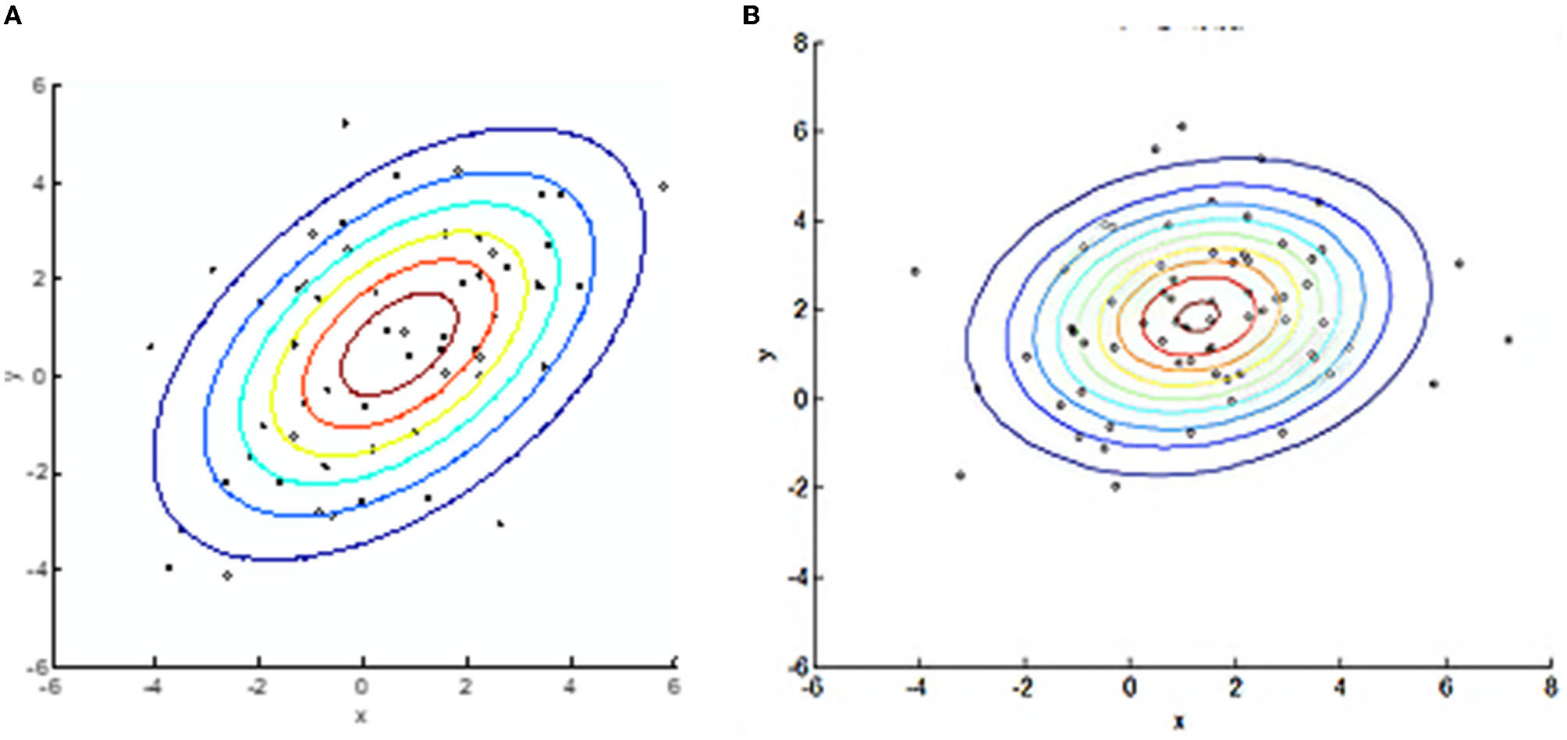
BEC kernel and Gaussian kernel with SVM for random nodes. **(A)** BEC kernel. **(B)** Gaussian kernel.

**Figure 6:**
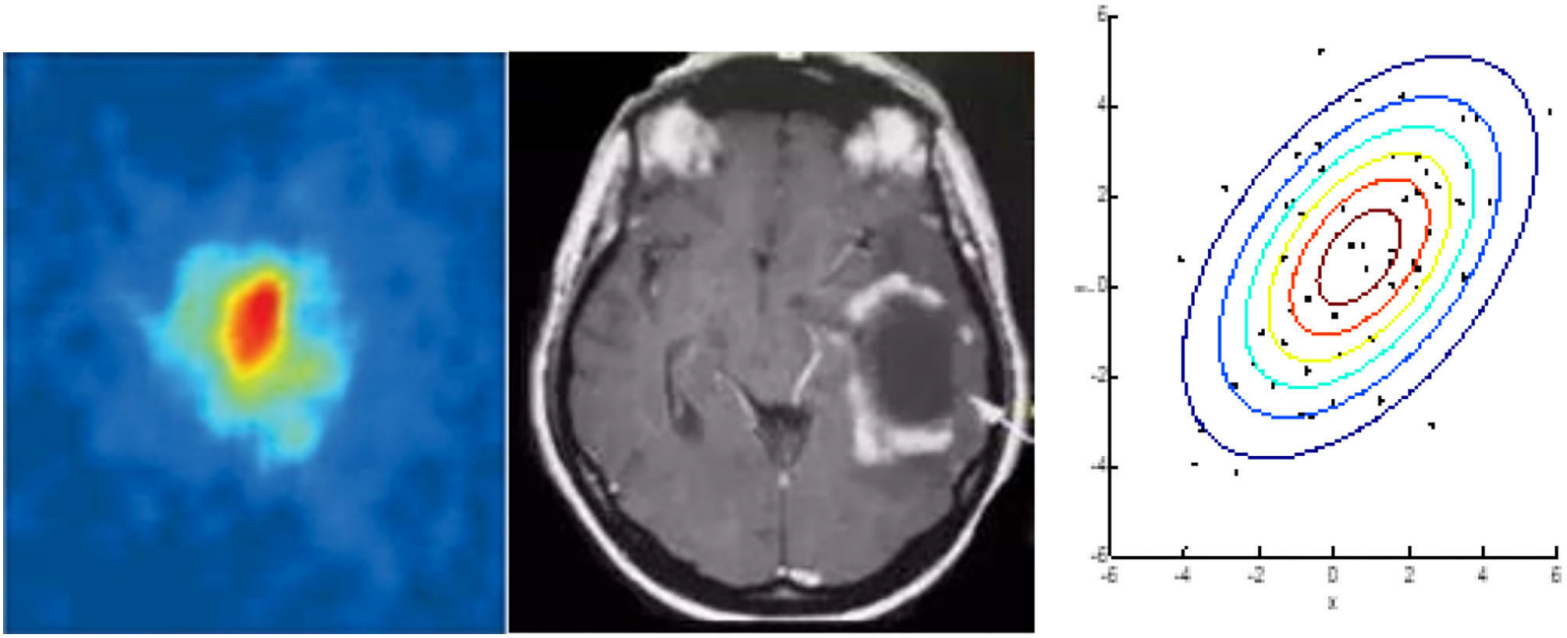
BEC image (Left), Glioma image (Middle), and BEC kernel in SVM (Right).

Figure 5A shows the segmentation performed by the BEC kernel of SVM (Support Vector Machine) for initially random nodes. Figure 5B highlights the segmentation produced by the Gaussian kernel of SVM for initially random nodes, and Figure 6 shows an image of Glioma (left), BEC image (middle), and the segmentation image by the BEC kernel of SVM (right). From these figures, we observed that the BEC kernel of SVM simulates the Glioma object better than the Gaussian kernel as the shape of the central circle of the BEC kernel looks more similar to the formation of glioma. Furthermore, the object in the glioma image, the BEC image, and the segmentation image by the BEC kernel all bear a similar underlying appearance of a central area with an outer annular region.

### Test 2: Nodes Clustered in Two Sets

**Figure 7:**
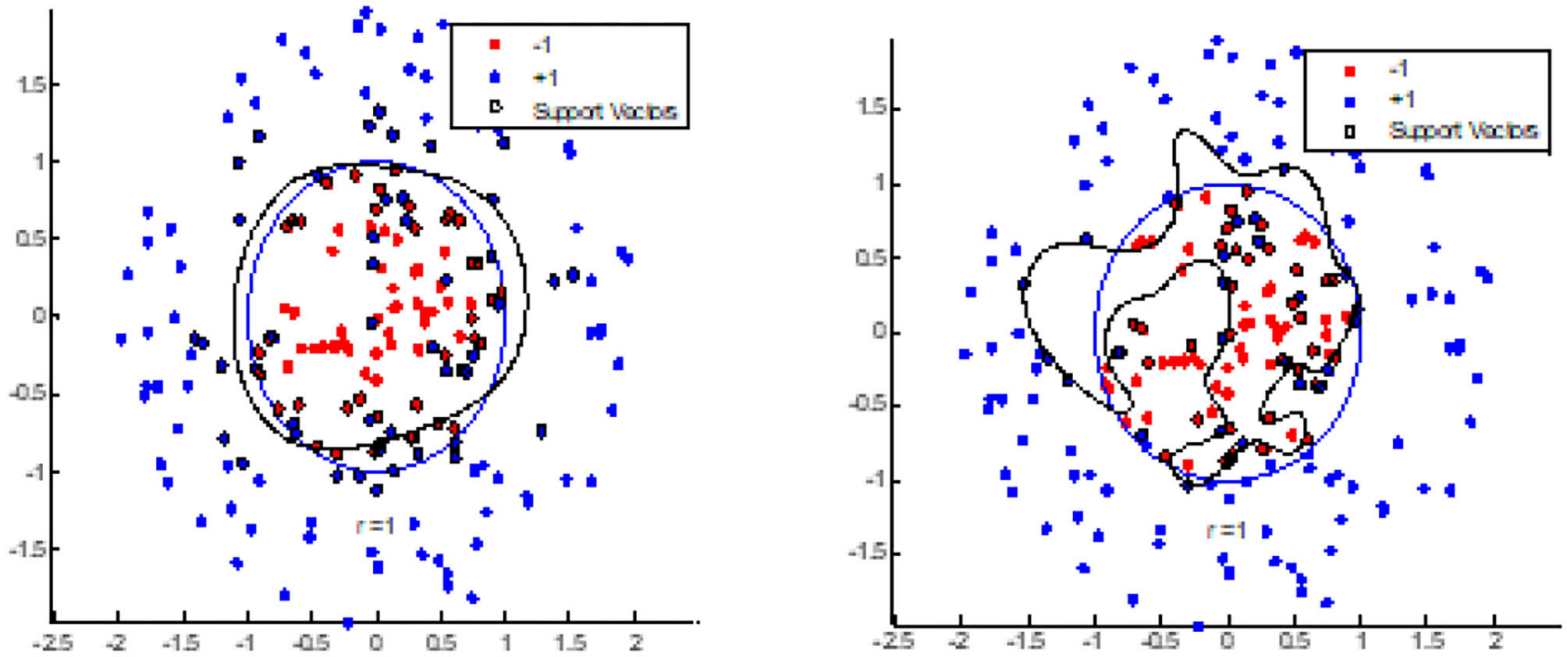
BEC kernel (Left) and Gaussian kernel (Right) for random nodes.

Test 2 is divided into 2 parts. In part one, we compared the BEC kernel of SVM and the Gaussian kernel of SVM to cluster two initially random node patterns. Figure 7 shows that the BEC kernel can cluster nodes more accurately when compared with the Gaussian kernel of SVM, as we see that the BEC kernel was able to press all the red-colored nodes while the Gaussian kernel did not.

**Figure 8:**
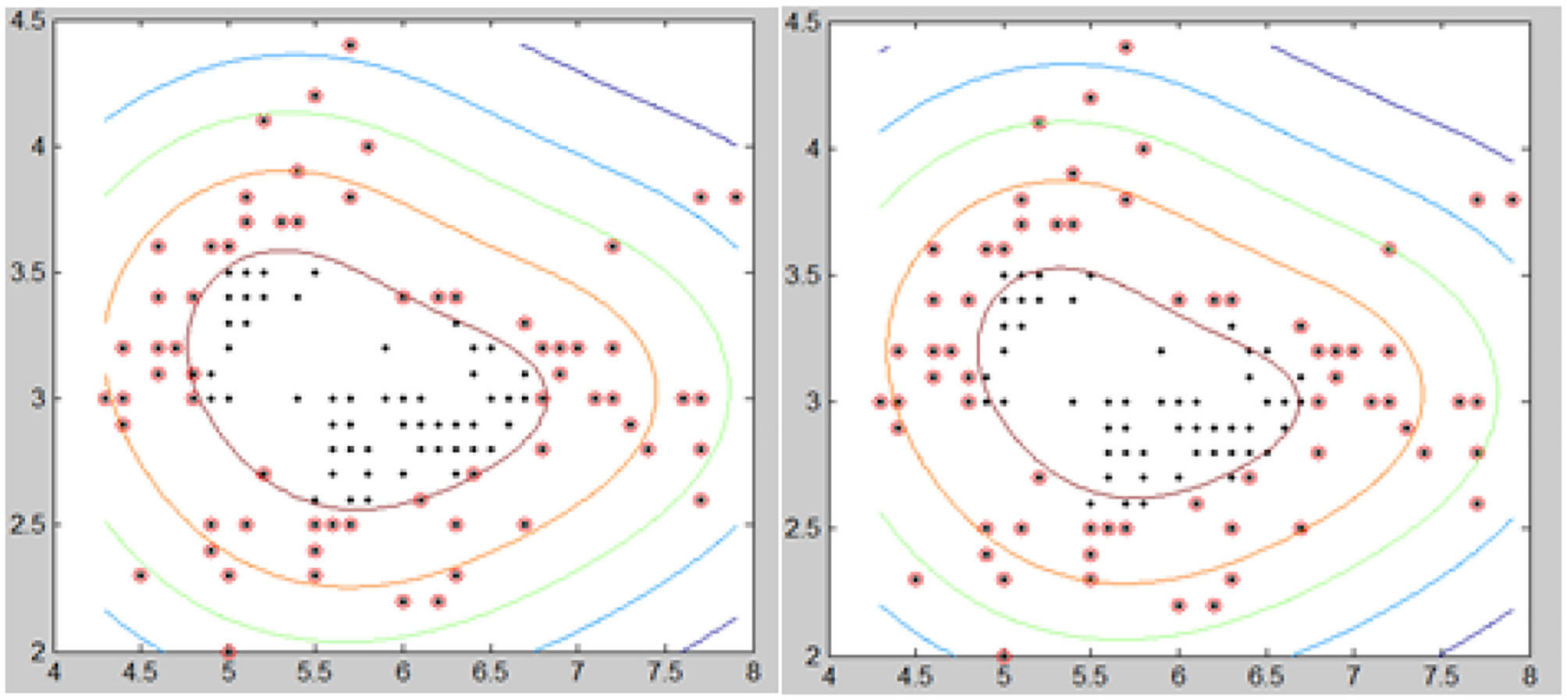
BEC kernel (Left) and Gaussian kernel (Right) for Fisheriris nodes.

The second part of test 2 compared the results obtained for the BEC kernel and Gaussian kernel using a Fisheriris node-set. Figure 8 shows that the clustering of the BEC kernel is more accurate than that of the Gaussian kernel as some of the nodes were not classified correctly by the Gaussian kernel.

Test 2 indicated that the BEC kernel of SVM performs better than a Gaussian kernel of SVM both in the case of an initially random node pattern and of a Fisheries node pattern set. It confirms that the BEC kernel is feasible in cluster images.

### Test 3: Image Segmentation of Glioma

Test 3 aimed to train the super-parameters of T and μ to investigate whether the BEC kernel can accurately segment a representative range of actual glioma types. We chose 18 glioma images in four groups of differing types from the MedPix® database, which included nearly 59,000 brain tumor images (https://medpix.nlm.nih.gov/home). These images were all typical glioma medical images, and the test results represented real-world segmentation performance. Group 1 was the type of image known as T^*^w^*^ and included four pictures. It was the typical type of glioma image, and we aimed to look for the best values for both T and μ. Group 2 consisted of six shots: Level I, Level II, Multiple gliomas T11, Multiple gliomas T12, Anaplastic oligodendroglioma I, and Anaplastic oligodendroglioma II. Group 2 was challenging for its complexity, and we aimed to find a trend of the best value for μ. Group 3 comprised of four T^*^ images of Oligodendrocytes where we aimed to find the movement for the best value of T. Group 4 contained images of Hemangiopericytoma (a) and Pleomorphic yellow astrocytoma (c). These were complicated gliomas, and we hoped to find the trend for best values of T and μ in these types. The range of T and μ was defined according to the occurrence of the BEC state. The value for T was lower than 0, but we aimed to trace the process of the BEC before and after the BEC state occurrence, so, the value range of T that we tested is slightly extended from −10 to 10. Similarly, the value range of μ was from −10 to 10 as well.

Image evaluation parameters P (Precision), R (Recall), and F (F-measure) were used to assess and compare the consistency, accuracy, and sensitivity, respectively (52), in the test results. Precision (P) was the fraction of relevant retrieved instances and determined how useful the results are: $\text{P}=\frac{\text{sum}\left(\text{predict}\;\&\text{true}\right)}{\text{sum}\left(\text{predict}\;\right)}$. Recall (R) Was the fraction of retrieved relevant instances and showed how complete the results are: $\text{R}=\frac{\text{sum}\left(\text{predict}\;\&\text{true}\right)}{\text{sum}\left(\text{true}\;\right)}$. The F-measure (F) is the harmonic mean of precision and recall. That is, $\text{F}=\frac{2*P*R}{\left(P+R\right)}$. A perfect image segmentation method should produce a Precision of 1, Recall of 1, and F-measure of 1. Meaning that all foreground pixels are correctly classified within tests, correctly classified across all the trials, and the measure approximately the average of the tw4o when they are similar.

#### Group 1

**Figure 9:**
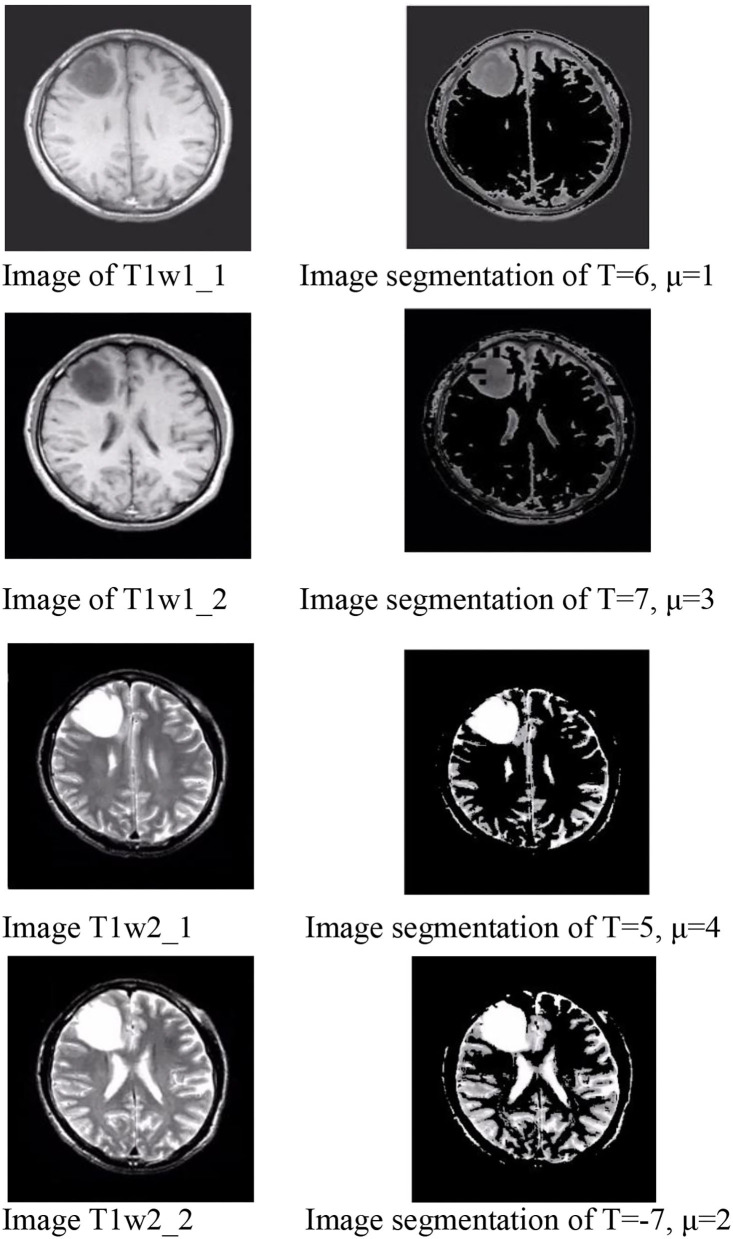
Image of group 1 and its best value image segmentation.

Group 1 included four types of images: type T1w1_1, T1w1_2, T2w1_1, and T2w1_2. For these glioma images, we tested the value of T from −10 to 10 and the range of μ from −10 to 10. The evaluation parameters of P, R, and F for group 1 are shown in Table 1. The best value of T and μ are T = 6 and μ = 1 for image T1W1_1, T = 7 and μ = 3 for image T1W1_2, T = 5 and μ = 4 for image T2W1_1, and T = −7 and μ = 2 for image T2W1_2. The segmentation images of the best values T and μ for group 1 are shown in Figure 9, and the values of T and μ are displayed in Table 1.

**Table 1:** Values of T and μ for group 1.

**T1W1_1**	**T5μ6**	**T5μ3**	**T-9μ3**	**T-9μ6**	**T6μ1**
R	0.9973	0.9973	0.9973	0.9973	0.9972
P	0.7731	0.7724	0.7708	0.7689	0.7567
F	0.8710	0.8705	0.8696	0.8683	0.8605
T1W1_2	T4μ3	T7μ3	T-7μ5	T4μ6	T7μ6
R	0.9972	0.9972	0.9972	0.9972	0.9972
P	0.7457	0.7446	0.7430	0.7383	0.7367
F	0.8533	0.8526	0.8515	0.8485	0.8474
T2W1_1	T5μ4	T-7μ5	T-5μ1	T5μ5	T-7μ3
R	0.9972	0.9972	0.9972	0.9972	0.9971
P	0.9104	0.9081	0.9048	0.8937	0.8845
F	0.9518	0.9505	0.9478	0.9426	0.9380
T2W1_2	T-7μ4	T-4μ3	T6μ6	T-7μ2	T-5μ6
R	0.9973	0.9973	0.9973	0.9973	0.9972
P	0.9083	0.9010	0.8923	0.8873	0.8715
F	0.9507	0.9467	0.9419	0.9391	0.9301

**Figure 10:**
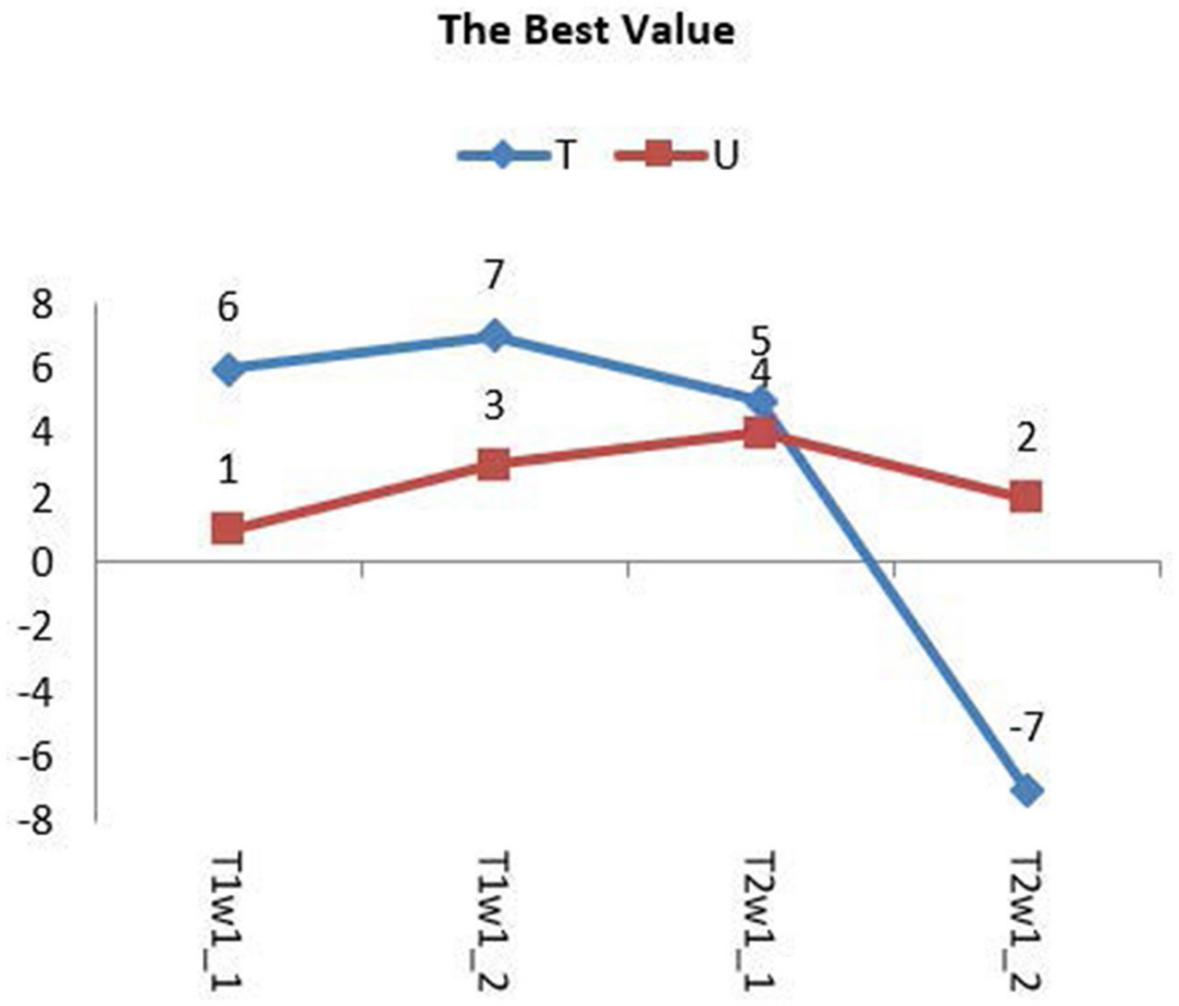
The tendency of best values T and μ for group 1.

The best values of T and μ for group 1 are shown in Figure 10. It shows that the best deals of T and μ are different for different types of gliomas image. However, they are all in the range from −7 to 7. The average best value of T is 6, and of μ is 3. Next, based on the average best value of T, we test the trend of the best value of μ in group 2 and then push the movement of the best value of T in group 3.

#### Group 2

**Figure 11:**
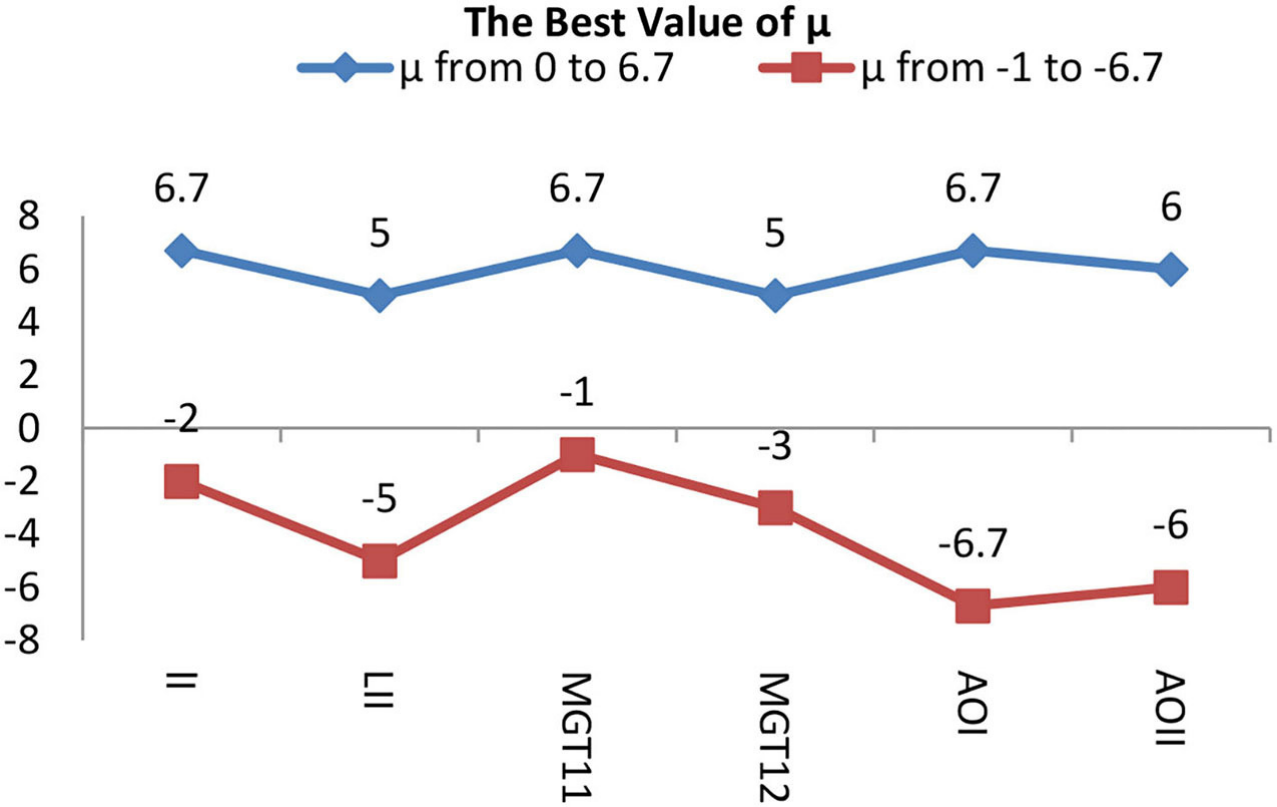
The best values for the six images of group 2.

In group 2, we focused on testing the trend for the best value of μ based on the best average value for T of 6 in group 1. This group included six images of three types, which have Levels [including Level I(LI) and Level II(LII)], multiple gliomas (including MG T11 and MG T12), and Anaplastic oligodendroglioma (including AOI and AOII). With these images shown by Figure 11, we test the value of μ, between 0 and 6.7, and from −1 to −6.7, while the value of T is 6. The test results are shown in Tables 2, 3. The best deal of μ is 6.7 and −2, −3 for Level I. The best value for μ is 5 and −5 for Level II. Values of μ from −6.7 to 6.7 are all good for Multiple gliomas T11. Values of μ from 2 to 5 and −3 to −5 are suitable for multiple gliomas T12. Values 6.7 and −6.7 are good for Anaplastic oligodendroglioma I (AOI). And, the values 6 and −6 are good values for Anaplastic oligodendroglioma II (AOII), as shown in Table 4.

**Table 2:** Image of group 2 in the range of μ was from 2 to 6.7.

**μ**	**2**	**3**	**4**	**5**	**6**	**6.7**	
LI	0.9961	0.9961	0.9962	0.9962	0.9961	0.9962	P
	0.8709	0.8552	0.8797	0.8818	0.8634	0.8854	R
	0.9293	0.9203	0.9343	0.9355	0.9250	0.9375	F
MG T11	**0.9963**	**0.9963**	**0.9963**	**0.9963**	**0.9963**	**0.9963**	P
	**1.000**	**1.000**	**1.000**	**1.000**	**1.000**	**1.000**	R
	**0.9981**	**0.9981**	**0.9981**	**0.9981**	**0.9981**	**0.9981**	F
AO I	0.9959	0.9959	0.9959	0.9959	0.9959	0.9961	P
	0.9151	0.9066	0.9075	0.9066	0.9130	0.9417	R
	0.9538	0.9491	0.9497	0.9491	0.9527	0.9681	F
MG T12	0.9961	0.9961	0.9961	0.9961	0.9961	0.9961	P
	1.000	1.000	1.000	1.000	0.9955	0.9936	R
	0.9981	0.9981	0.9981	0.9981	0.9958	0.9949	F
L II	0.9962	0.9962	0.9962	0.9962	0.9962	0.9962	P
	0.9891	0.9962	0.9968	1.000	0.9943	0.9818	R
	0.9926	0.9962	0.9965	0.9981	0.9952	0.9889	F
AO II	0.9952	0.9952	0.9952	0.9952	0.9952	0.9952	P
	0.9939	0.9915	0.9922	0.9918	0.9946	0.9900	R
	0.9945	0.9933	0.9937	0.9935	0.9949	0.9926	F

*The bold words mean that the value is good and corresponds to a value that does not change for different parameters*.

**Table 3:** Image of group 2 of the range of μ was from −2 to −6.7.

**μ**	**−2**	**−3**	**−4**	**−5**	**−6**	**−6.7**	
L I	0.9962	0.9962	0.9961	0.9961	0.9961	0.9961	P
	0.8818	0.8818	0.8619	0.8749	0.8645	0.8746	R
	0.9335	0.9335	0.9242	0.9316	0.9256	0.9314	F
MG T11	**0.9963**	**0.9963**	**0.9963**	**0.9963**	**0.9963**	**0.9963**	P
	**1.000**	**1.000**	**1.000**	**1.000**	**1.000**	**1.000**	R
	**0.9981**	**0.9981**	**0.9981**	**0.9981**	**0.9981**	**0.9981**	F
AO I	0.9959	0.9959	0.9959	0.9959	0.9959	0.9961	P
	0.9075	0.9066	0.9075	0.9066	0.9130	0.9417	R
	0.9497	0.9491	0.9497	0.9491	0.9527	0.9681	F
MG T12	0.9961	0.9961	0.9961	0.9961	0.9961	0.9961	P
	0.9974	1.000	0.9955	1.000	1.000	0.9936	R
	0.9968	0.9981	0.9958	0.9981	0.9981	0.9949	F
L II	0.9962	0.9962	0.9962	0.9961	0.9962	0.9962	P
	0.9928	0.9962	0.9962	0.9869	0.9951	1.000	R
	0.9945	0.9962	0.9962	0.9915	0.9956	0.9981	F
AO II	0.9952	0.9952	0.9952	0.9952	0.9952	0.9952	P
	0.9899	0.9919	0.9897	0.9926	0.9915	0.9922	R
	0.9925	0.9935	0.9924	0.9939	0.9934	0.9937	F

*The bold words mean that the value is good and corresponds to a value that does not change for different parameters*.

**Table 4:** Image of group 2 of the best value of μ was from −6.7 to 6.7.

**Name of image**	**μ**	**μ**
level 1 (LI)	6.7	−2 and −3
level II (LII)	5	−5
Multiple gliomas (MG) T11	0 to 6.7	−1 to −6.7
Multiple gliomas (MG) T12	2 to 5	−3 and −5
Anaplastic oligodendroglioma (AO)I	6.7	−6.7
Anaplastic oligodendroglioma (AO)II	6	−6

**Figure 12:**
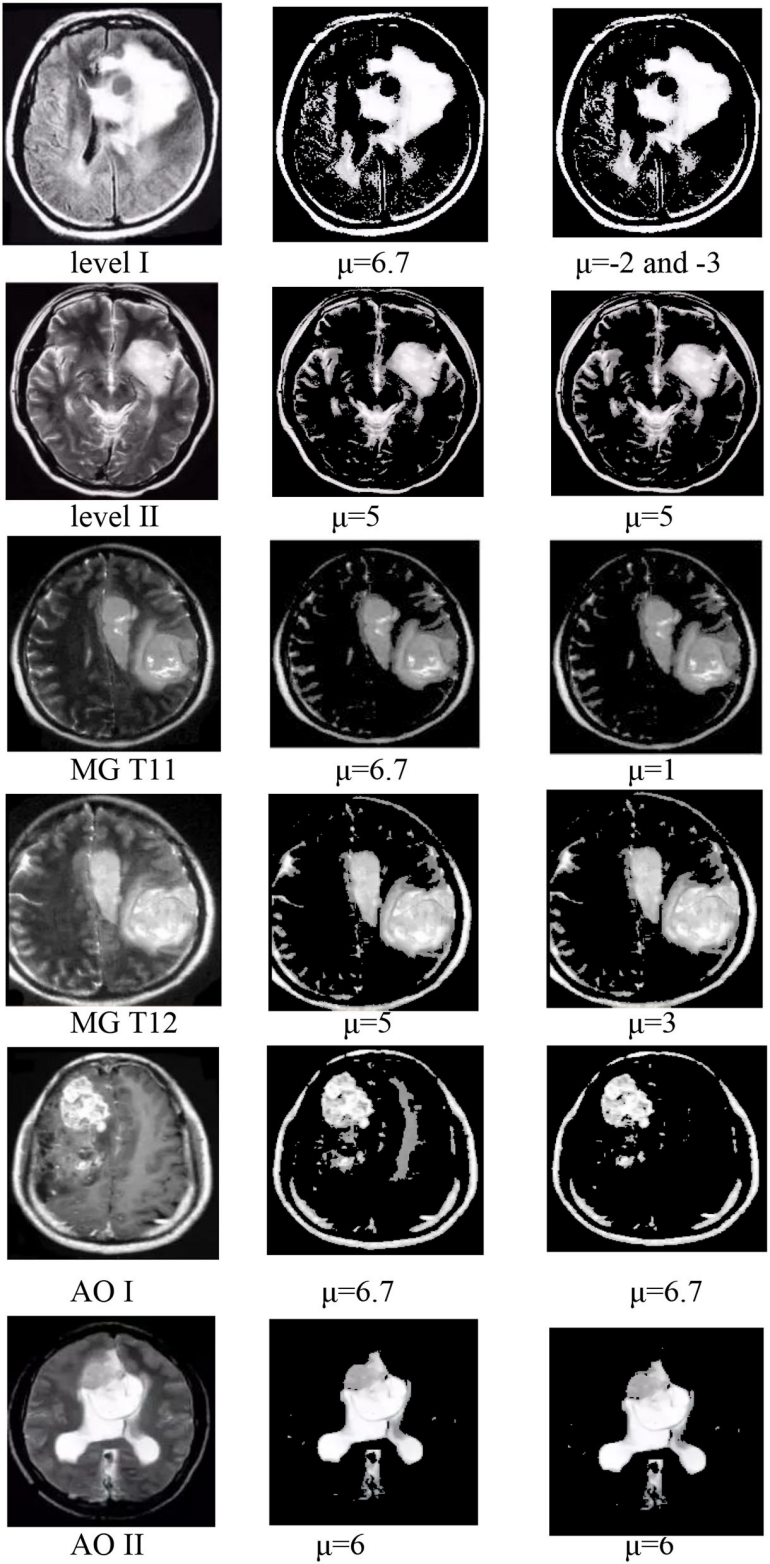
Image of group 2 and its best value segmentation.

The trend of the best value of μ is shown in Figure 12. It shows that the importance of μ has a slight change for different types. Still, the positive and negative values of μ have the same trend, except for the AOI image, and they almost have a symmetric relationship for each image, except for the appearance of L I. The conclusion is that we can use the positive values of μ in place of the negative values of μ when segmentation is not required, and the best value range of μ is from 5 to 6.7.

#### Group 3

**Figure 13:**
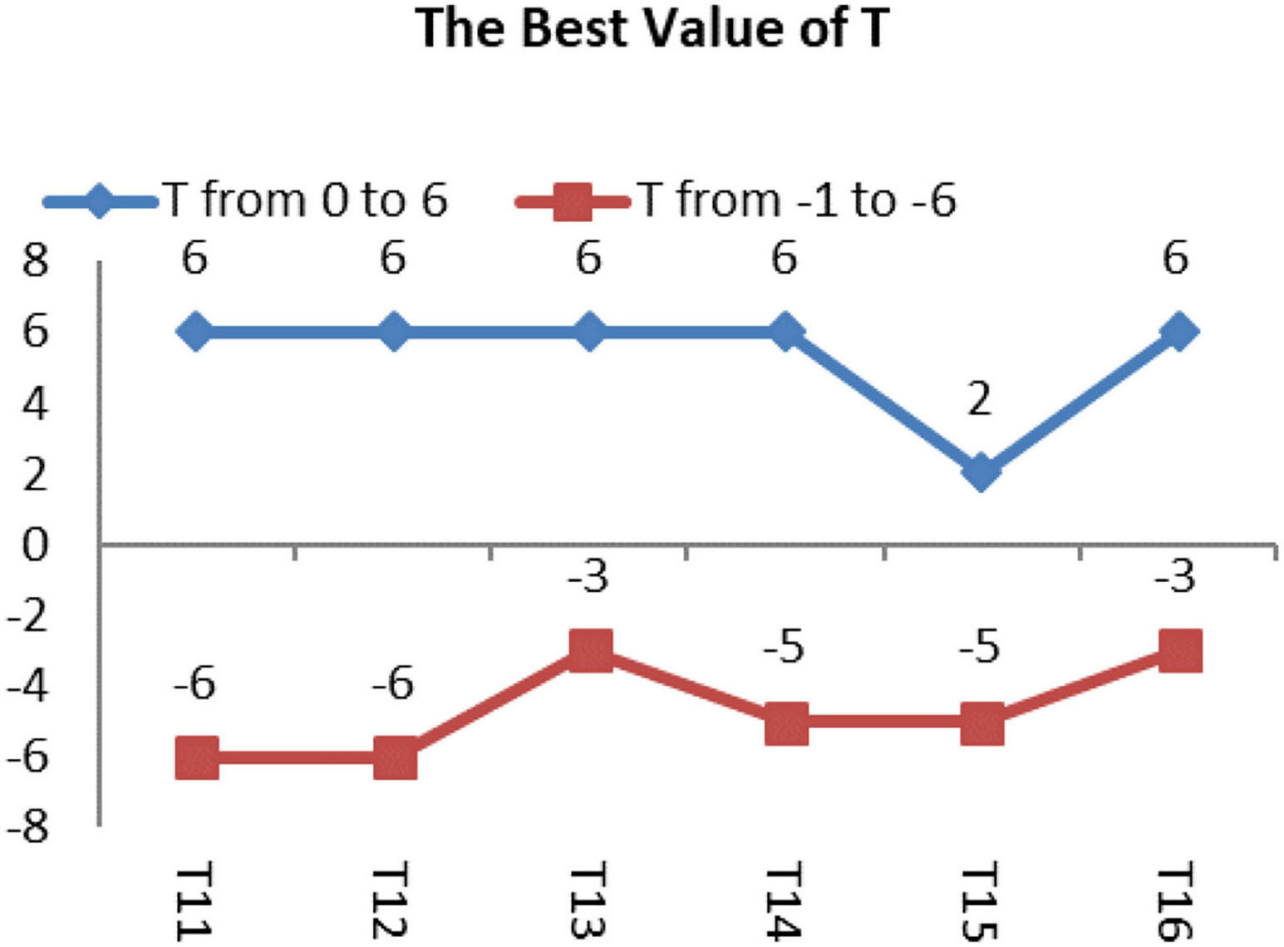
Shows the image segmentation of the best value of T for group 3.

**Figure 14:**
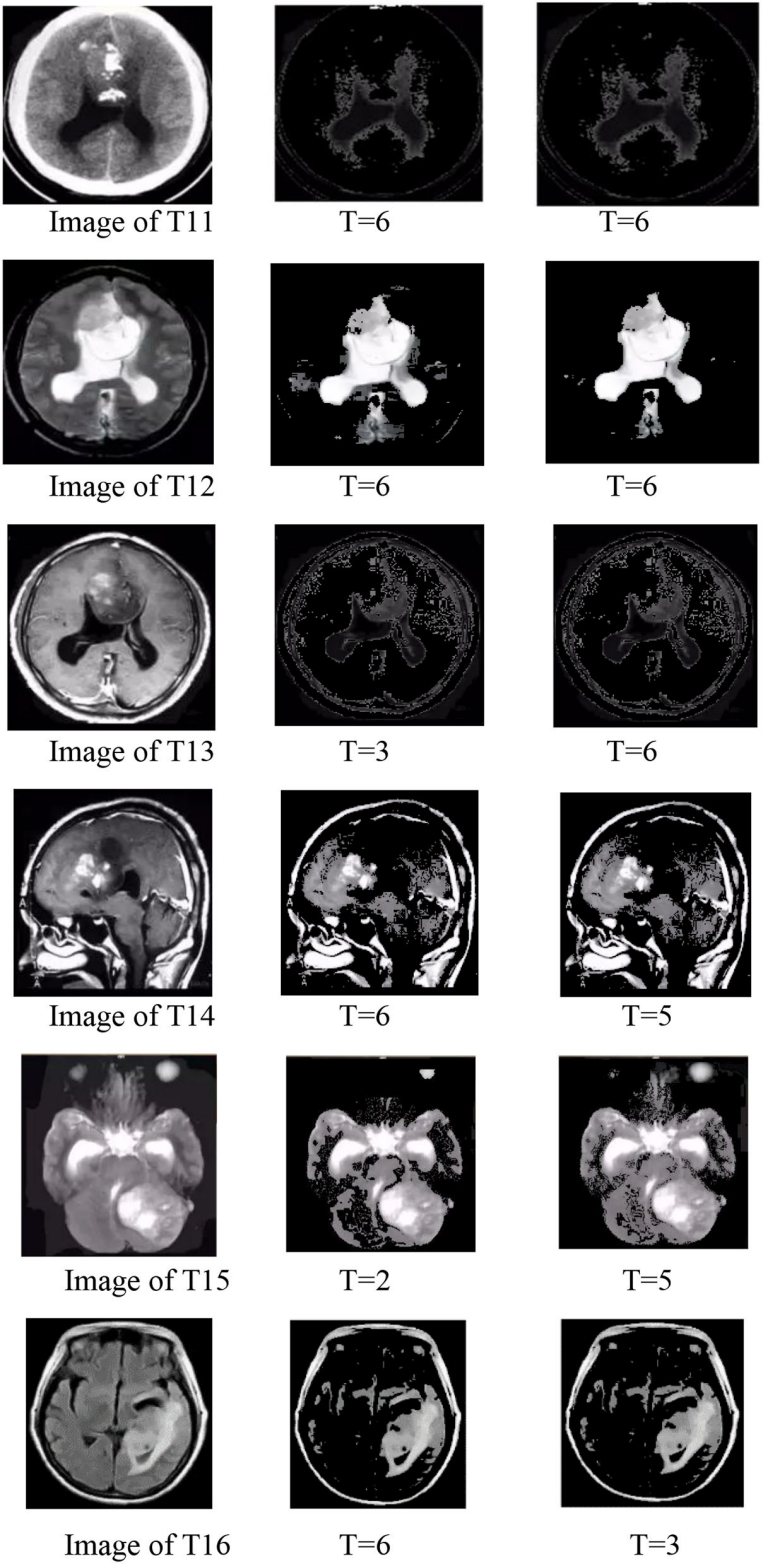
Images of group 3 and their best value of T.

The best value of *T test* is shown by Figure 13, and the Images shown by Figure 14 in group 3 were Oligodendrocytes and included the T11, T12, T13, T14, T15, and T16. We concentrated on testing the trend for the best value of T based on the best average value of μ of 3 in group 1. We tried the T value in the BEC formula from 0 to 6 and from **–**1 to −6. The results are shown in Tables 5, 6. Values of T from 0 to 6 were all good for T11 and T13. The best deal of T was 6 for T12. A T value of 0, 2, and 6 was suitable for T14, and a T value of 2 was ideal for T15. T values of 5, 6 were ideal for T16, and a T value of −6 was good for T11 and T13, while a T value of −3 was good for T13 and T16, and T value of −5 was good for T14 and T15.

**Table 5:** Image of group 3 within range of T is from 0 to 6.

**T**	**0**	**1**	**2**	**3**	**5**	**6**	
T11	**0.9945**	**0.9945**	**0.9945**	**0.9945**	**0.9945**	**0.9945**	P
	**0.8507**	**0.8507**	**0.8507**	**0.8507**	**0.8508**	**0.8507**	R
	**0.9170**	**0.9170**	**0.9170**	**0.9170**	**0.9170**	**0.9170**	F
T12	0.9952	0.9952	0.9952	0.9952	0.9952	0.9952	P
	0.9923	0.9877	0.9896	0.9919	0.9898	0.9943	R
	0.9937	0.9914	0.9924	0.9935	0.9915	0.9974	F
T13	**0.9948**	**0.9948**	**0.9948**	**0.9948**	**0.9948**	**0.9948**	P
	**0.8492**	**0.8492**	**0.8492**	**0.8492**	**0.8492**	**0.8492**	R
	**0.9612**	**0.9612**	**0.9612**	**0.9612**	**0.9612**	**0.9612**	F
T14	0.9955	0.9954	0.9955	0.9954	0.9955	0.9955	P
	1.0000	0.9893	1.0000	0.9835	0.9999	1.0000	R
	0.9977	0.9924	0.9977	0.9894	0.9977	0.9977	F
T15	0.9966	0.9966	0.9966	0.9966	0.9965	0.9966	P
	0.9815	0.9927	0.9944	0.9886	0.9538	0.9815	R
	0.9890	0.9946	0.9955	0.9926	0.9747	0.9890	F
T16	0.9961	0.9961	0.9961	0.9961	0.9962	0.9962	P
	0.9905	0.9827	0.9827	0.9719	1.0000	1.0000	R
	0.9933	0.9893	0.9893	0.9838	0.9981	0.9981	F

*The red color indicates the best value and blue color indicates next best value for different parameters that do not change*.

**Table 6:** Image of group 3 within range of T is from −1 to −6.

**T**	**–1**	**−2**	**−3**	**−4**	**−5**	**−6**	
T11	**0.9945**	**0.9945**	**0.9945**	**0.9945**	**0.9945**	**0.9945**	P
	**0.8508**	**0.8507**	**0.8507**	**0.8507**	**0.8508**	**0.8512**	R
	**0.9170**	**0.9170**	**0.9170**	**0.9170**	**0.9170**	**0.9173**	F
T12	0.9952	0.9952	0.9952	0.9952	0.9952	0.9952	P
	0.9898	0.9920	0.9903	0.9900	0.9921	0.9936	R
	0.9925	0.9936	0.9928	0.9926	0.9936	0.9944	F
T13	**0.9948**	**0.9948**	**0.9948**	**0.9948**	**0.9948**	**0.9948**	P
	**0.8492**	**0.8492**	**0.8514**	**0.8492**	**0.8492**	**0.8492**	R
	**0.9162**	**0.9162**	**0.9175**	**0.9162**	**0.9162**	**0.9162**	F
T14	0.9954	0.9954	0.9954	0.9953	0.9955	0.9955	P
	0.9935	0.9910	0.9954	0.9744	1.000	0.9911	R
	0.9945	0.9932	0.9954	0.9848	0.9977	0.9933	F
T15	0.9966	0.9965	0.9965	0.9965	0.9966	0.9966	P
	0.9785	0.9606	0.9738	0.9592	0.9995	0.9785	R
	0.9875	0.9782	0.9850	0.9775	0.9981	0.9875	F
T16	0.9961	0.9961	0.9962	0.9960	0.9961	0.9961	P
	0.9936	0.9877	1.000	0.9701	0.9905	0.9798	R
	0.9948	0.9919	0.9987	0.9874	0.9933	0.9879	F

*The red color indicates the best value and blue color indicates next best value for different parameters that do not change*.

We concluded that it is possible to include the positive values of T instead of the negative values of T, if specific segmentation is not required, and the best value range for T is from 2 to 6.

#### Group 4

**Figure 15:**
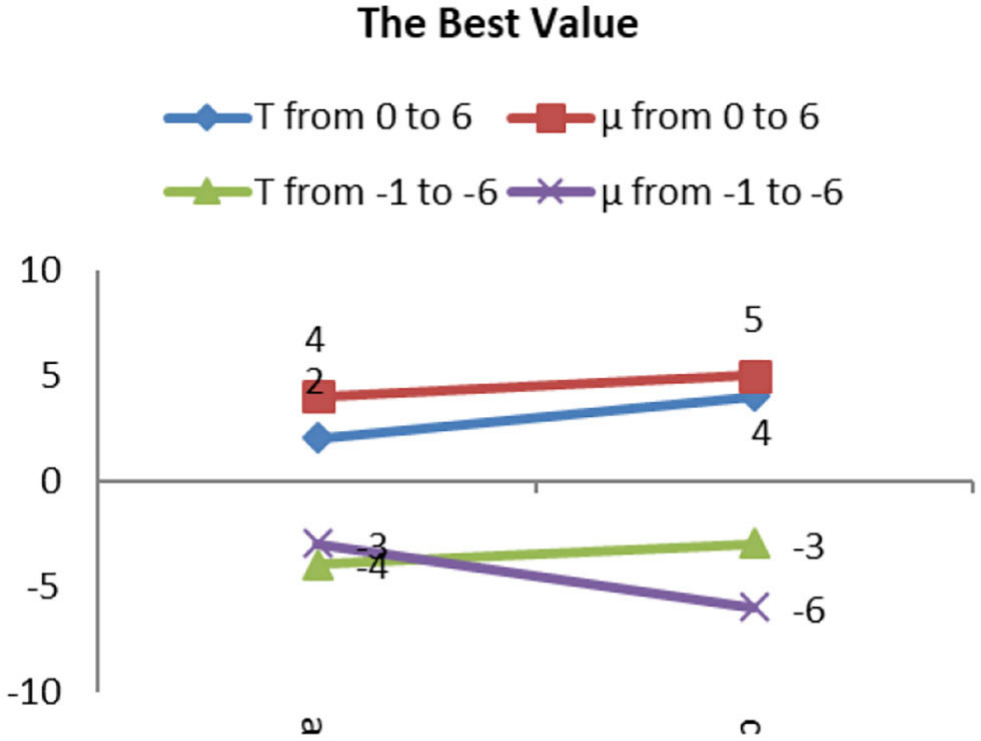
Best values for the two images of group 4.

**Figure 16:**
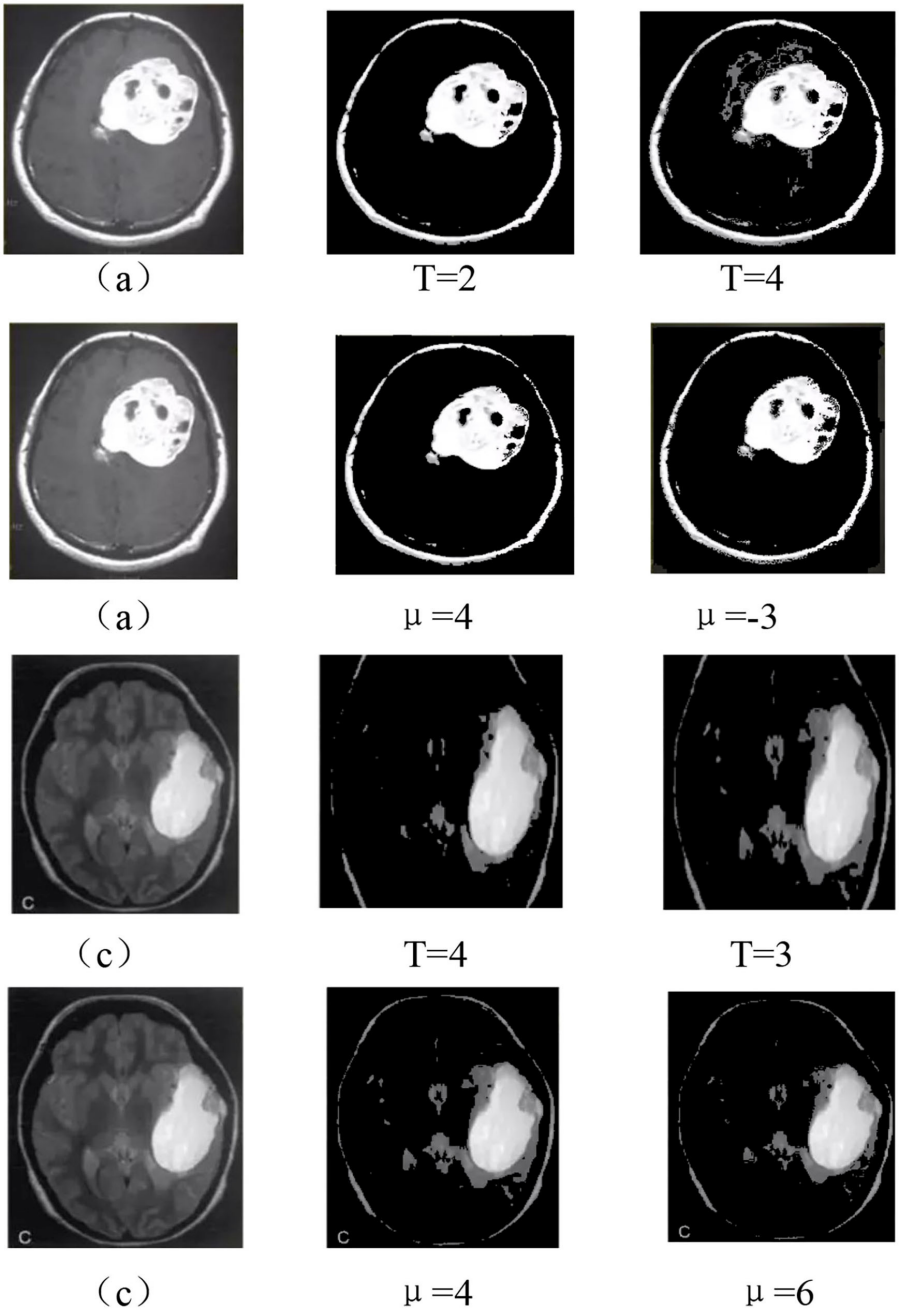
Image group 4 and its best value segmentation image.

In group 4, we focused on attempting to detect the best values for both T and μ based on the value trends in the testing of group 2 and group 3. Group 4 images shown by Figure 15 included two images: Hemangiopericytoma (a) and Pleomorphic yellow astrocytoma (c). The changing trend of the values of T and μ is shown in Figure 16. The best values in group 4 had the same trend as group 1, group 2, and group 3.

The value range of T and μ were from −6 to 6, and as shown by Table 7; we noticed that the best T value was 2 for image (a), and 1, 3, 4 for the image of (c). The best value for μ was 4 for image (a), and 1 and 5 for the appearance of (c). From Table 8, we observed that the best T value was −4 for image (a) and −1 and −3 for (c). The best μ value was −3 for (a), −3, −4, −5, and −6 for the image of (c). Table 7 shows the detailed test parameters of T and μ.

**Table 7:** Image of group 4.

**T, μ**	**1**	**2**	**3**	**4**	**5**	**6**
**T(a)**						
P	0.9967	0.9967	0.9967	0.9967	0.9967	0.9967
R	0.9600	0.9688	0.9646	0.9658	0.9635	0.9658
F	0.9780	0.9826	0.9804	0.9810	0.9798	0.9810
**T(c)**						
P	0.9961	0.9961	0.9961	0.9961	0.9961	0.9961
R	1.000	0.9928	1.000	1.000	9952	0.9958
F	0.9980	0.9944	0.9980	0.9980	0.9956	0.9959
**μ (a)**						
P	0.9967	0.9967	0.9967	0.9967	0.9967	0.9967
R	0.9637	0.9637	0.9648	0.9680	0.9536	0.9633
F	0.9799	0.9799	0.9805	0.9821	0.9747	0.9797
**μ (c)**						
P	0.9961	0.9961	0.9961	0.9961	0.9961	0.9961
R	0.9929	0.9967	0.9996	0.9997	1.000	0.9957
F	0.9945	0.9964	0.9978	0.9979	0.9980	0.9959

*The range of T and μ was from 0 to 6. The red color indicates the best value and blue color indicates next best value for different parameters that do not change*.

**Table 8:** Image of group 4.

**T, μ**	**−1**	**−2**	**−3**	**−4**	**−5**	**−6**
**T(a)**						
P	0.9966	0.9967	0.9967	0.9967	0.9967	0.9967
R	0.9515	0.9616	0.9702	0.9767	0.9650	0.9650
F	0.9735	0.9788	0.9833	0.9866	0.9806	0.9806
**T(c)**						
P	0.9961	0.9961	0.9961	0.9961	0.9961	0.9961
R	1.000	0.9888	1.000	0.9948	0.9979	0.9941
F	0.9980	0.9924	0.9980	0.9954	0.9970	0.9951
**μ (a)**						
P	0.9967	0.9967	0.9967	0.9967	0.9967	0.9966
R	0.961	0.9611	0.9689	0.9645	0.9650	0.9535
F	0.9785	0.9812	0.9826	0.9803	0.9805	0.9746
**μ (c)**						
P	0.9961	0.9961	0.9961	0.9960	0.9961	0.9961
R	0.9988	0.9998	1.000	0.9917	0.9998	0.9999
F	0.9974	0.9979	0.9980	0.9939	0.9980	0.9980

*The range of T and μ was from −1 to −6. The red color indicates the best value and blue color indicates next best value for different parameters that do not change*.

We concluded that the best value of T and μ are within the range that we predicted at the beginning of the tests.

In summary, from the above eighteen tests, which were divided into four groups of glioma images, we conclude that the range of the values of T and μ are from −10 to 10, which is larger than the standard range of T ≤ 0 and μ from 0 to 6.7 as suggested by the underlying theory of the BEC approach. Our test aimed to investigate whether the range of values would be more extensive or if it included any unexpected results. However, the results indicate a performance as predicted by calculation. In addition, the best values that we tested are all within the range of the BEC theory. Therefore, the four groups of 18 glioma images proved that our proposed prediction model with the BEC kernel is feasible and has a good performance for glioma image segmentation.

### Test 4: Comparison of Results With Other Similar Methods

In this test, we compared our prediction model with the existing cluster reference models and methods in the field of brain image segmentation of about seven years, from the year 2014 to 2020 of reference (52–61), such as Gray-Level Co-occurrence Matrix (GLCM), large margin local estimate (LMLE), Capturing statistic (C.P.) + principal component analysis (PCA) and Histogram-gravitational optimization (HbGO), Gaussian Mixture Model, Disentanglement and Gated Fusion, and some improved fuzzy clustering, namely, entropy-based fuzzy, improved fuzzy clustering, and FCM+rough set. The mean comparison result showed that our prediction model using the BEC kernel provided higher evaluation values in Precision, Recall, and F-measure. For example, for the value of P, the best value among reference methods was 0.98, while for our process, all were more than 0.99. As for the value of R, the highest value of our BEC kernel approach was 1 while the existing approaches had a value of 0.96. Moreover, the lowest R-value for our method was better than the six reference papers except the three papers related to GLCM, LMLE, and HbGO. Finally, our comprehensive value F, which included P and R values, was better than all the methods except for the Khan and al. approach (53), in which the F value was between 0.88 and 0.93. Similarly, our approach returns values that were between 0.85 and 0.99, which were a little bit similar or higher. In the other cases, our F values were better than the existing approaches. Therefore, we can conclude that our prediction model using the BEC kernel had significantly achieved better results than the existing methods. Table 9 shows a comparison of methods.

**Table 9:** Our prediction model compared with reference methods.

**Authors**	**Method**	**Precision** **(mean)**	**Recall** **(mean)**	**F- (mean)**
Khan and Syed (53)	GLCM	0.94–0.98	0.92–0.96	0.88–0.93
Song et al. (54)	LMLE	0.73–0.90	0.80–0.87	0.76–0.88
Erus et al. (55)	CP +PCA	0.99	0.46–0.62	0.53–0.66
Nabizadeh et al. (56)	HbGO	0.82–0.89	0.75–0.92	0.70–0.86
Prakash and Kumari (57)	Gaussian Mixture Model	0.90–0.94	0.72–0.82	0.80–0.87
Kahali etal. (58)	entropy-based fuzzy	0.78–0.82	0.68–0.70	0.72–0.76
Ren etal. (59)	improved fuzzy clustering	0.88–0.90	0.71–0.80	0.78–0.85
Huang et al. (60)	FCM + rough set,	0.95	0.70–0.81	0.80–0.87
Chen etal. (61)	Disentanglement and Gated Fusion	0.80–0.85	0.73–0.89	0.76–0.87
Our model	BEC kernel	0.99	0.73–1.00	0.85–0.99

### Test 5: Comparison of Results With CNN Methods Based on Both BraTS Datasets and Clinical Images

Since CNN has been an innovative technology in recent years, we added a comparative experiment with it. We used the challenging datasets BraTS and clinical images for the segmentation experiment to confirm the effectiveness of the prediction model proposed in this article.

For verifying the effectiveness of the method, two groups of medical images with different datasets were further selected for experiments: BraTS (the Brain Tumor Segmentation) Datasets and clinical patents. Group 1 of BraTS datasets is used for standard and challenging testing, and clinical patent datasets of group 2 aimed at testing the practical effect. Group 1 images were selected from BraTS 2020, 2019, and 2018 datasets, each with modality volume of 240 × 240 × 155. Each sample was composed of four modalities of brain MRI scans T1-weighted (T1), post-contrast T1-weighted (T1ce), T2-weighted (T2), and Fluid Attenuated Inversion Recovery (FLAIR). Group 2 consisted of 100 patient images which were selected from the clinical, radiological, and hospital cases. The selected 100 tumor MRI images included grades II, III, and IV with an image size of 256 × 256 × 3.

#### Group 1

**Figure 17:**
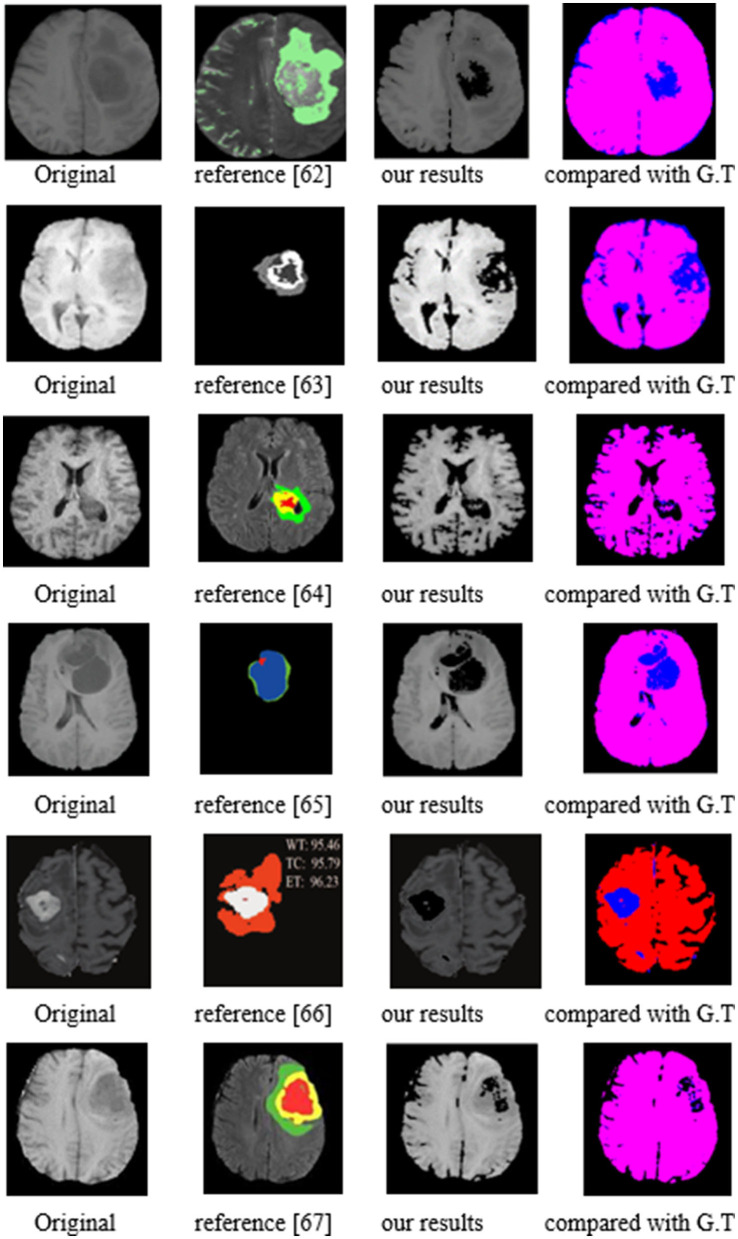
The segmentation of the image results of Brain Tumor Segmentation (BraTS).

From BraTS2020, 2019, and 2018 datasets, we chose 150 cases of the training sample, 50 cases for validation, and 25 cases of test samples. The test segmentation examples are shown by Figure 17 with the compared reference results (62–67). The evaluated parameters are shown in Table 10.

**Table 10:** The evaluated segmentation results of Brain Tumor Segmentation (BraTS) datasets.

	**Ma et al. (62), Ours**	**Zhou et al. (63), Ours**	**Wang et al. (64), Ours**	**Wu et al. (65), Ours**	**Liu et al. (66), Ours**	**Zhou et al. (67), Ours**	**Our model**
P	0.900, 0.9967	0.9159, 0.9545	0.964–0.968, 0.9978	0.906, 0.9878	0.993–0.997, 1.0000	0.870–0.962, 0.9945	0.9545–1.0000
R	0.850, 0.9138	0.8274, 0.8584	–, 0.9463	0.950, 0.8887	0.807–0.924, 0.952–0.9836	–, 0.9461	0.8584–0.9836
F	0.870, 0.9535	0.8694, 0.9039	–, 0.9714	0.928, 0.9356	0.948–0.9591, 0.975–0. 9917	–, 0.9697	0.9039–0.9917

In Figure 17, the first column represents the original image, while the second column contains the reference segmentation results. The third one is our segmentation results, and the fourth column is our results compared with ground truth (G.T.). The red color area is the G.T. results, while the blue one is our segmented results. The overlapping of the blue and red zones means that the segmented results are accurate. As for the images compared with G.T., we can notice that the red and blue areas almost overlap, indicating that our segmentation is accurate. When compared with the reference images, our segmentation contours are closer to the target area than the reference images. The following segmentation has evaluated the parameters that further prove the accuracy of our approach.

The reference papers in Table 10 represent the existing CNN methods in glioma segmentation from 2018 to 2021. Concerning the three evaluated parameters, P, R, and F, our values are all higher than other approaches using the same images. As an example, in the best-compared value by Liu and et al. (66), published in 2021, their value of P is from 0.993 to 0.997, but ours is 1. In addition, their R value ranged from 0.807 to 0.924, while our R value is between 0.9523 and 0.9836. Notably, our lowest value, 0.9523, is higher than their highest value of 0.924. Even more, when comparing the value of F, our lowest value is 0.975, which is also higher than their highest value of 0.9591.

#### Group 2

**Figure 18:**
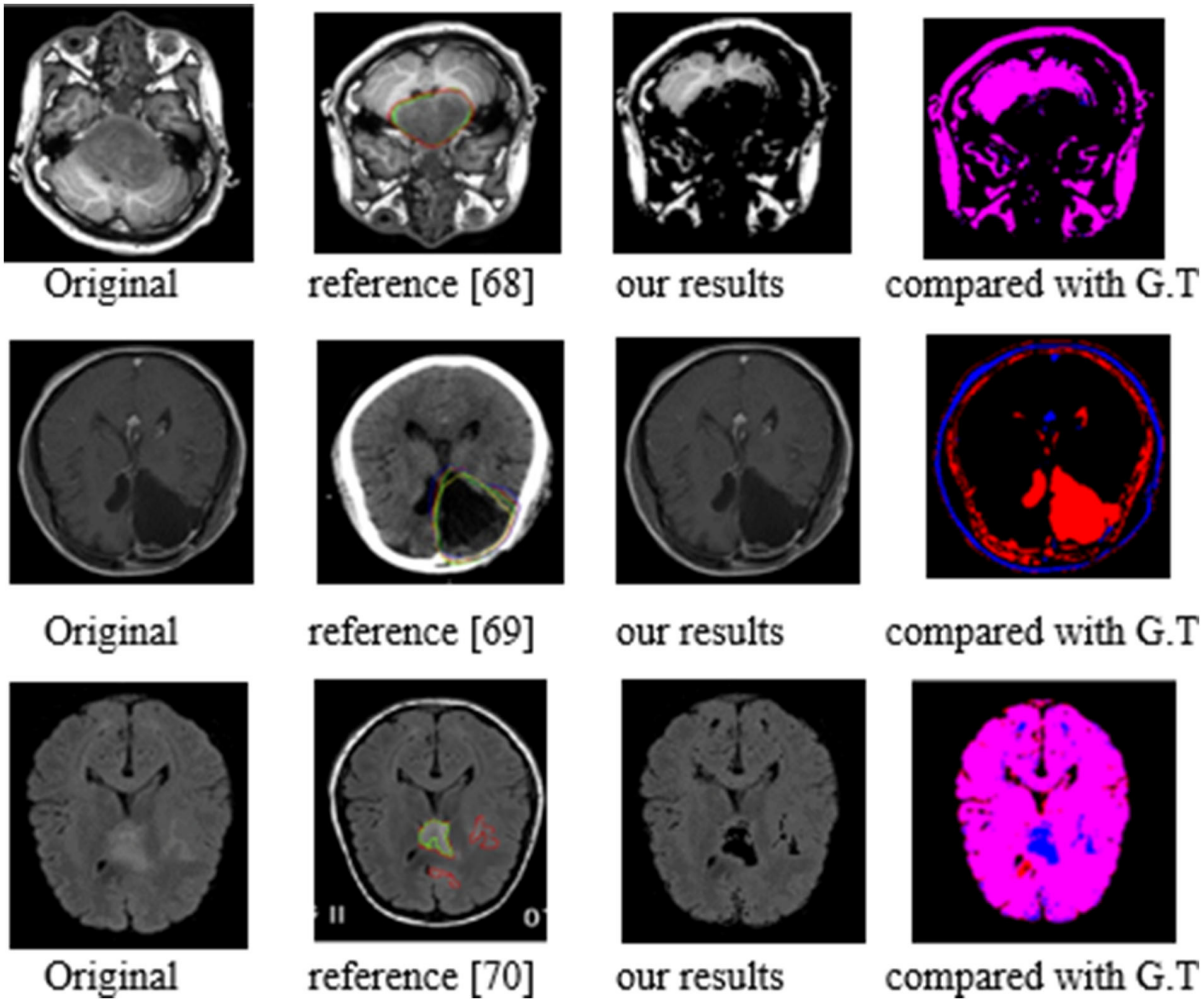
The example of patient images segmentation results.

Around 100 patient images were collected from 55 patients. The MRI and CT images were collected from Beijing Tiantan Hospital of China and were randomly divided into two sets with 38 patients as training set, four patients as validation set, and 13 patients as testing set (68). Forty-five patient cases were from the clinical, radiological tumor images dataset (69, 70). The examples from 100 test case results are shown in Figure 18 compared to the reference results, and the evaluation parameters are indicated in Table 11.

**Table 11:** The evaluated segmentation results of patient images.

	**Jia et al. (68), Ours**	**Tang et al. (69), Ours**	**Naser and Deen (70), Ours**	**Our prediction model**
P	0.9652, 1.0000	0.933, 0.9961	0.89–0.92, 0.9623	0.9623–1.0000
R	0.9667, 0.9643	0.993, 0.9672	0.87–0.92, 0.9469	0.9469–0.9672
F	0.9659, 0.9818	0.962, 0.9814	0.88–0.92, 0.9545	0.9545–0.9818

As shown in the second and third columns, our segmentation contours are closer to the target contours. Our results presented in the four-column images reveal an overlap between red and blue areas, which confirms the accuracy of our segmentation contours. The following evaluation parameters table proves that our method results are better than existing methods.

Regarding the images of clinic patients, all our test values of P, R, and F are higher than the values of existing methods. For example, in the best test values of the approach of Liu and et al. (68), *P*-value is 0.9652, R-value is 0.9667, and F is 0.9659. When implementing the same test, P and F values of our method are harmonic as P and R of 1 and 0.9818. It means that our prediction model has better test values than the existing models.

Both group 1 and group 2 test results demonstrated that the proposed prediction model in this article has more accurate segmentation in BraT datasets and has a comparative validity in clinic patient datasets.

## Conclusions

This article presents a new prediction model using the BEC kernel to improve the glioma image segmentation. The proposed BEC kernel is based on the quantum theory of the BEC state model, a novel approach that was not previously explored for image segmentation. First, the BEC theory was analyzed to illustrate that applying the BEC kernel is feasible and potentially advantageous for the image segmentation. Second, we formulated a novel prediction model for image segmentation, based on a BEC kernel. Third, a BEC kernel, which was innovatively derived from quantum mechanics theory and other related research, was used. Finally, experiments were conducted using both BraTS Datasets and clinical images to validate our proposed prediction mode. In conclusion, the proposed prediction model segmentation is more accurate than the existing cluster methods and the most commonly used CNN techniques for implementing brain image segmentation tasks.

As a future work, we plan to investigate the problem of medical image segmentation through the proposed prediction model for more different types of medical images, especially for tumors apart from brain tumors. In addition, we are planning to test more theories in the quantum mechanics research field and apply them in medical image segmentation.

## Data Availability Statement

Publicly available datasets were analyzed in this study. This data can be found here: https://mp.weixin.qq.com/s/d1H2j5vjrVo-RHRAVI8ygQ; https://www.med.upenn.edu/cbica/brats2020/data.html; https://www.med.upenn.edu/cbica/brats2019/data.html; https://www.med.upenn.edu/sbia/brats2018/data.html.

## Author Contributions

TZ designed the method, conducted the experiments, and wrote the manuscript. SC handled this project. JZ helped to improve the results and modified the manuscript. BS revised the manuscript and figures and enhanced their overall quality, and also improved the MATLAB scripts used in this analysis. Finally, all authors read this revised manuscript and gave final approval for the final submission.

## Funding

This research was supported by the 2020-2022 National Natural Science Foundation of China under Grand (Youth) No. 52001039, 2020-2022 Funding of Shandong Natural Science Foundation in China, No. ZR2019LZH005, and 2022-2025 National Natural Science Foundation of China under Grand, No. 52171310.

## Conflict of Interest

The authors declare that the research was conducted in the absence of any commercial or financial relationships that could be construed as a potential conflict of interest.

## Publisher's Note

All claims expressed in this article are solely those of the authors and do not necessarily represent those of their affiliated organizations, or those of the publisher, the editors and the reviewers. Any product that may be evaluated in this article, or claim that may be made by its manufacturer, is not guaranteed or endorsed by the publisher.

## Footnotes

^1^ https://en.wikipedia.org/wiki/Glioma

^2^ http://www.jupiterscientific.org/sciinfo/boseeinstein.html

^3^ https://en.wikipedia.org/wiki/Bose%E2%80%93Einstein_condensate

^4^ https://figshare.com/articles/Diffuse_Low_grade_Glioma_Database
